# Nutrition Controls Mitochondrial Biogenesis in the *Drosophila* Adipose Tissue through Delg and Cyclin D/Cdk4

**DOI:** 10.1371/journal.pone.0006935

**Published:** 2009-09-09

**Authors:** Claudia Baltzer, Stefanie K. Tiefenböck, Mark Marti, Christian Frei

**Affiliations:** 1 Department of Biology, ETH Zurich, Zurich, Switzerland; 2 PhD program in Molecular Life Sciences, Zurich, Switzerland; Roswell Park Cancer Institute, United States of America

## Abstract

Mitochondria are cellular organelles that perform critical metabolic functions: they generate energy from nutrients but also provide metabolites for de novo synthesis of fatty acids and several amino acids. Thus mitochondrial mass and activity must be coordinated with nutrient availability, yet this remains poorly understood. Here, we demonstrate that *Drosophila* larvae grown in low yeast food have strong defects in mitochondrial abundance and respiration activity in the larval fat body. This correlates with reduced expression of genes encoding mitochondrial proteins, particularly genes involved in oxidative phosphorylation. Second, genes involved in glutamine metabolism are also expressed in a nutrient-dependent manner, suggesting a coordination of amino acid synthesis with mitochondrial abundance and activity. Moreover, we show that Delg (CG6338), the *Drosophila* homologue to the alpha subunit of mammalian transcription factor NRF-2/GABP, is required for proper expression of most genes encoding mitochondrial proteins. Our data demonstrate that Delg is critical to adjust mitochondrial abundance in respect to Cyclin D/Cdk4, a growth-promoting complex and glutamine metabolism according to nutrient availability. However, in contrast to nutrients, Delg is not involved in the regulation of mitochondrial activity in the fat body. These findings are the first genetic evidence that the regulation of mitochondrial mass can be uncoupled from mitochondrial activity.

## Introduction

In eukaryotes, cellular organelles are separated from the cytoplasm through lipid membranes, creating compartments with unique biological properties. Rather than static, organelle size and function are often dynamic, and tightly regulated in response to various stimuli. One of the best-studied organelles are mitochondria, which show large cell-type specific variations in morphology and abundance, demonstrating that mitochondria are highly regulated. Mitochondrial dysfunction is linked to various diseases, including metabolic disorders and cellular aging [1], therefore these organelles are critical for cellular homeostasis. Mitochondria perform multiple metabolic functions, most notably the generation of energy from carbohydrates, fatty acids and amino acids. Equally important, mitochondria also provide metabolites for anabolic processes such as *de novo* synthesis of fatty acids and amino acids. Although the metabolic biochemical reactions are well established, we are just beginning to understand how these processes are coordinated *in vivo*. One interesting question is how nutrients control mitochondrial mass and activity, and how this regulation affects cellular metabolism.

During cellular growth, amino acids are used for protein synthesis. In higher eukaryotes, essential amino acids are taken up through the diet, whereas nonessential amino acids are synthesized *de novo*. For the latter, mitochondria are critical, since they provide oxaloacetate for aspartate and asparagine, as well as 2-oxoglutarate (α-ketoglutarate) for glutamate, glutamine, arginine and proline biosynthesis. Of these amino acids, glutamine is particularly interesting: First, many cell types take up large amounts of glutamine, which can be used to produce cytoplasmic NAD^+^, NAPDH and lactate in a process called glutaminolysis. Second, since the TCA cycle intermediate citrate can be used as a substrate for fatty acid synthesis, glutamine can be converted into 2-oxoglutarate, thus replenishing the TCA cycle. Third, efflux of cytoplasmic glutamine, either taken up or synthesized *de novo*, is directly linked to the uptake of essential amino acids, both in mammals and *Drosophila* [2]. Interestingly, all three processes are highly active in cancer cells, under conditions of high metabolic activity [3]. One would therefore expect tight coordination between nutrients, mitochondrial activity and amino acid synthesis, in particular glutamine, yet factors mediating such links have not been described.

Mitochondria contain their own genome (mtDNA), encoding a small number of proteins required for oxidative phosphorylation (OXPHOS), as well as tRNAs and rRNAs for mitochondrial translation. The majority of mitochondrial proteins are encoded by the nuclear genome, including factors for mitochondrial transcription and translation. These proteins are translated in the cytoplasm and imported into mitochondria. Accordingly, the transcription of these nuclear genes is believed to be rate limiting for mitochondrial mass and activity [4]. To understand the nutrient-specific regulation of mitochondria, one has to characterize how these nuclear transcription factors are regulated in response to nutrients. In *Drosophila*, genes encoding mitochondrial proteins are highly expressed during the larval growth and feeding period. Subsequently, as the larvae stop feeding at the end of the last larval instar and prepare for metamorphosis, expression of these genes is strongly downregulated [5], [6]. Thus *Drosophila* larval growth is an ideal system to study how mitochondria are regulated in response nutrients *in vivo*. This has been exploited in recent microarray studies, where expression profiles of normal fed and starved larvae were compared: Indeed, starvation led to a strong downregulation of genes involved in mitochondrial translation, respiration, TCA cycle, fatty acid oxidation and mitochondrial transport [7], [8]. Similar findings have been published using microarrays from fed or starved adult flies [9]. Comparing larval fat body and muscle tissues, Teleman et al. discovered that many of these genes respond in a cell-type specific manner [8]. Therefore, factors must exist that mediate a tissue-specific transcriptional control in response to nutrients. One candidate for such a factor is dFoxo, the fly homologue to mammalian forkhead O-type transcription factors (FoxO family) [10]. Importantly, dFoxo did only mediate the nutrient responsiveness for a subset of genes encoding mitochondrial proteins [8]. This implies that other transcription factors must exist, yet they have not been described in *Drosophila*.

In this study, we used *Drosophila melanogaster* to characterize mitochondria in a developing organism *in vivo*. We focused on the larval fat body, the fly adipose/liver tissue. Fat body cells are specified during embryogenesis, and show an enormous increase in cell size during larval stages, which is accompanied by endoreduplication of its DNA to a C-value of ∼256. Growth of these cells is directly regulated by nutrient uptake [11], making the larval fat body an ideal system to study mitochondria in response to nutrition and nutrient-sensitive growth-promoting pathways. We show that low-yeast food conditions, and thus amino acid starvation, leads to strongly reduced mitochondrial abundance and respiration activity. This correlates with reduced expression of genes encoding mitochondrial proteins, including enzymes involved in glutamine metabolism. Moreover, *Drosophila* Delg (CG6338), the fly homologue to the alpha subunit of mammalian transcription factor NRF-2/GABP, functions as a key regulator for mitochondrial mass. Surprisingly, reduced mitochondrial mass in *delg* mutants does not translate into reduced OXPHOS activity. Rather, residual mitochondria compensate by being more active. More importantly, our data show that Delg is critical to adjust mitochondrial abundance and expression levels of enzymes required for glutamine metabolism in response to nutrient availability. Finally, we observed that the nutrient-sensitive growth-promoting complex Cyclin D/Cdk4 required Delg for its effect on mitochondria. Thus our data demonstrate how Cyclin D/Cdk4 and Delg coordinate mitochondrial abundance and glutamine metabolism with nutrient availability *in vivo*.

## Results

### Mitochondria in the larval fat body respond to low-yeast nutrition

**Figure 1:**
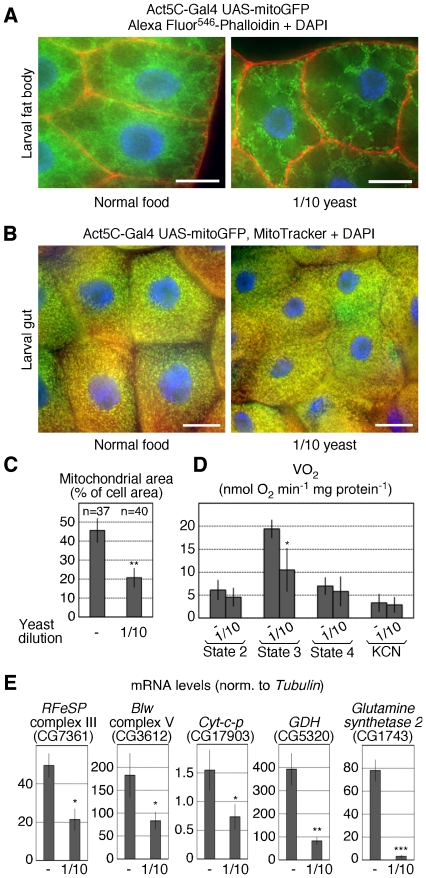
Nutrient-specific control of mitochondrial abundance and activity in the larval fat body. Larvae were grown in formal food, or in food that contained 10% of the normal yeast concentration. (A) Mitochondrial-targeted GFP (mitoGFP) was expressed using the Act5C-Gal4/UAS system, and the cell-outline was stained using phalloidin (shown in red). Shown is the larval fat body. (B) MitoGFP was expressed as in A, and the tissue was counterstained using the mitochondrial-specific dye MitoTracker. Shown is the larval midgut, close to the proventiculus. (C) Quantitation of A. Mitochondrial area per cell was measured using ImageJ. Analysis was done in blind. (D) Oxygen consumption assays on digitonin-permeabilized dissected fat bodies. State 2 to 4 respirations were measured after the serial addition of pyruvate and proline (state 2), ADP (state 3) and the ADP/ATP transporter inhibitor atractyloside (state 4). Respiration was normalized to total fat body protein. Averages and standard deviations are shown from 4 biological replicates. (E) Transcript levels using qPCR from dissected fat bodies, normalized to *Tubulin* levels. Shown are levels for genes involved in mitochondrial respiration (*RFeSP*, *Blw* and *Cytochrome-c-proximal*), as well as *Glutamate dehydrogenase* (*GDH*) and *Glutamine synthetase 2*, encoding enzymes involved in glutamate and glutamine biosynthesis. A to E: Mid-third instar control larvae (Df(3R)ro^80b^/+) were taken 4 days AED. Bar equals 20 µm.

Standard *Drosophila* food contains high amounts of carbohydrates (sugar and corn) and yeast, the latter providing mostly amino acids and fatty acids, but also compounds for which flies are auxotrophic. To test whether mitochondrial abundance is regulated in response to nutrients, we grew larvae in normal or low-yeast food, and ubiquitously expressed a mitochondrial-targeted GFP (mitoGFP) to label mitochondria. Since low-yeast food leads to a delay in development, mid-third instar larvae were taken for all experiments except when indicated (4 days after egg deposition (AED) for normal fed, and 8 days AED for low-yeast fed control larvae). When analyzed in the larval fat body, mitochondria were abundant throughout the cytoplasm in normal food. In contrast, when larvae were grown under low-yeast conditions (10% of normal levels), the mitoGFP signal was dispersed and less abundant in the cytoplasm (Fig. 1A). Since average cell size was not changed (data not shown), we labeled the plasmamembrane with phalloidin, and quantified the GFP-positive area and normalized it to the cell area. Under normal food conditions, mitochondria occupied 45% of the cell area. This was significantly reduced to 21% under low yeast food (Fig. 1C). To confirm that mitoGFP labels mitochondria, we compared its fluorescence with MitoTracker, a mitochondrial-specific dye. Indeed, the two fluorescences perfectly overlapped (Fig. S1A), demonstrating that mitochondrial abundance is regulated in response to nutrients. To test whether this effect is specific for the fat body, we analyzed other larval tissues. In the gut, using both mitoGFP and MitoTracker, mitochondria were equally abundant under both feeding conditions (Fig. 1B). Other larval tissues did also not show apparent changes in abundance upon low-yeast food (data not shown). Taken together, these data demonstrate a tissue-specific control of mitochondrial abundance in response to nutrients. Given that the fat body is critical for the whole animal to respond to amino acid availability [12], we focused on this tissue to further characterize the effect of nutrients on mitochondria.

Mitochondrial oxidative phosphorylation generates ATP from NADH and FADH_2_, which are derived from the mitochondrial TCA cycle or during fatty acid oxidation. During this process, electrons from NADH and FADH_2_ are transferred to the electron transport chain, which uses the electron-transfer potential to create a proton gradient across the inner-mitochondrial membrane. ATP synthase then generates ATP from ADP and phosphate.

Oxygen serves as the final electron acceptor for the electron transport chain, thus oxygen consumption reflects OXPHOS activity. To test whether the reduced mitochondrial abundance under low-yeast conditions would result in oxidative phosphorylation defects, we adapted an assay for mammalian tissue samples to measure fat body-specific oxygen consumption [13]. We dissected larval fat bodies, permeabilized the plasmamembrane with digitonin, and measured oxygen consumption using a Clark electrode. When normalized to protein content, the addition of pyruvate and proline (state 2), which stimulates complex I of the electron transport chain, led to identical respiration rates when larvae were grown in normal or low-yeast food (Fig. 1D). Under these conditions, ADP is limiting. Therefore, after the addition of ADP (state 3), fat bodies from normal fed larvae showed a 3.2-fold increase in respiration rates. In contrast, this was significantly reduced to 2.3-fold when fat bodies were dissected from low-yeast fed larvae, demonstrating respiration defects under these conditions (Fig. 1D). In addition to the generation of ATP, the inner-mitochondrial proton gradient is dissipated through uncoupling complexes, producing heat. In mammals, two adipose tissues are found: The white adipose tissue, which mediates energy storage, and the brown adipose tissue, which shows a high degree of uncoupling, being involved in thermongenesis. It is not known whether the fly fat body functions as a white or brown adipose tissue. To measure uncoupled respiration, we used atractyloside, an inhibitor of the ADP/ATP transporter, leading to ADP depletion (state 4). We did not detect different respiration rates between normal and low-yeast fed animals (Fig. 1D). Furthermore, when respiration was measured in two other larval tissues, the gut and the epidermis, the ratios between state 3 and state 4 respiration were not changed compared to the fat body under normal feeding conditions (data not shown). We conclude that first, uncoupled respiration is not nutrient-responsive. Second, the fly fat body resembles the mammalian white adipose tissue. Finally, we added KCN to block all mitochondrial respiration by inhibiting complex IV of the electron transport chain. We did not detect a difference between fat bodies from normal and low-yeast fed larvae (Fig. 1D). Taken together, mitochondrial abundance in the larval fat body is reduced under low-yeast food conditions, leading to respiration defects.

As mentioned above, many genes encoding mitochondrial proteins are transcriptional regulated in response to nutrients in the larval fat body [8]. To test whether reduced expression of OXPHOS genes could explain the respiration defects, we used quantitative PCR (qPCR) to measured mRNA levels from dissected fat bodies. Indeed, *RFeSP*, encoding the Rieske iron-sulfur protein of complex III, *Bellwether* (*Blw*), encoding the ATP synthase subunit alpha protein of complex V and *Cyct-c-p*, encoding Cytochrome c, were all expressed at significantly reduced levels under low yeast food (Fig. 1E). Next, we measured mRNA levels of genes encoding enzymes involved in the synthesis of glutamate, glutamine and proline, which are all synthesized from the TCA cycle intermediate 2-oxoglutarate (see below for a schematic representation of these enzymatic reactions). Of 8 genes encoding these enzymes, 6 were significantly downregulated upon low-yeast food, including *Glutamate dehydrogenase* (*GDH*; conversion of 2-oxoglutarate and glutamate), *Glutamine synthetase 2* (conversion of glutamate and glutamine), and *Pyrroline-5-carboxylate reductase* (conversion of glutamate and proline; Fig. 1E, 7C and S1B). These data demonstrate that nutrients must control transcription factors that drive the expression of genes encoding mitochondrial proteins. In addition, this suggests that the glutamine metabolism is coordinated with mitochondrial abundance and activity.

### *Delg* mutants have reduced mitochondrial mass in the *Drosophila* larval fat body

**Figure 2:**
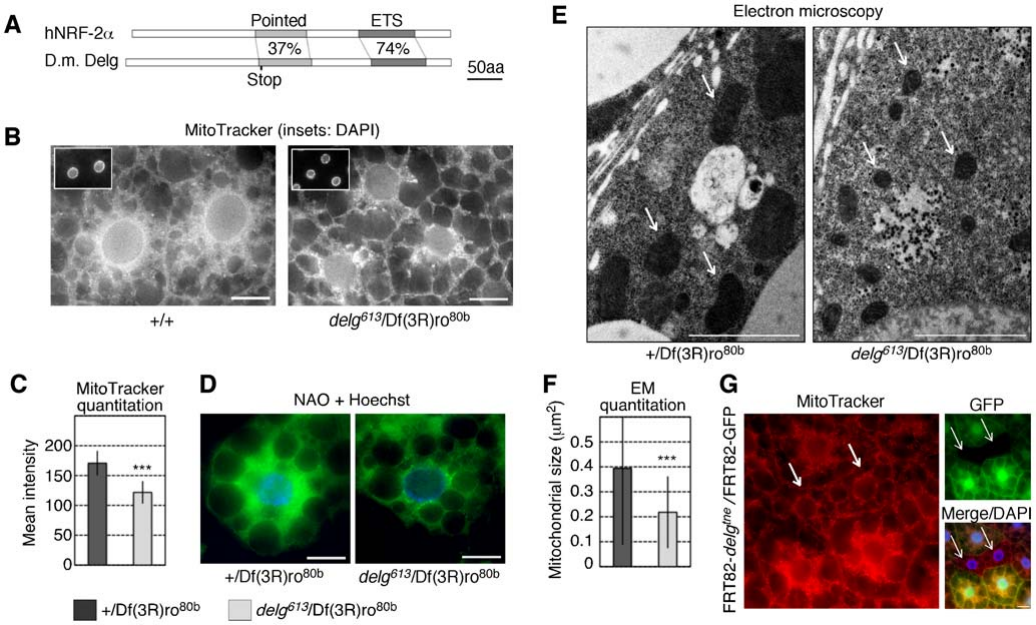
The *Drosophila* NRF-2α/GABPβ homologue Delg is required for mitochondrial biogenesis. (A) Schematic representation of the homology between human NRF-2α and *Drosophila* Delg. The pointed and ETS domains are 37% and 74% identical in amino acid sequence, respectively. The Q^193^ to Stop codon mutation in *delg^613^* [21] is indicated. (B) MitoTracker and DAPI stainings of fixed fat bodies from third instar larvae. Whereas wild type cells show abundant staining throughout the cytoplasm, *delg* mutant cells show a strong reduction in staining. (C) Quantitation of MitoTracker staining. Mean fluorescent intensity per cell was calculated in ImageJ. Quantitation was done in blind, n≥21. (D) Nonylacridine orange (NAO; in green), which detects the mitochondrial phospholipid cardiolipin, and Hoechst^33342^ (specific for DNA, shown in blue) stainings in unfixed larval fat bodies. (E) Electron microscopy images of larval fat bodies. Arrows point to mitochondria, which show a strong size reduction in *delg* mutant cells. (F) Quantitation of electron microscopy, shown is the average mitochondrial area, n≥34. (G) Homozygous mutant clones for *delg* were induced using the Flp/FRT system, *delg^tne^/delg^tne^* cells are recognized by the absence of GFP (arrows), and show strong, cell-autonomous reduction in MitoTracker staining. Bar equals 20 µm, except 2 µm in E.

In mammals, several transcription factors are known to mediate the expression of genes encoding mitochondrial proteins, including NRF-1 and NRF-2/GABP (nuclear respiratory factor-1 or -2/guanine adenine binding protein) [14], [4]. To test whether their fly homologues could mediate the nutrient-specific regulation in the fat body described above, we tested available mutants for mitochondrial defects in the larval fat body. *Drosophila* Ewg (Erect wing) is the only NRF-1 homologue, and functionally important in neurons and muscle tissues [15]. We induced homozygous mutant clones in the larval fat body as shown below for Fig. 2G of an *ewg* null allele (*ewg^2^*), but did not observe any mitochondrial phenotypes when assayed using MitoTracker (data not shown). NRF-2 is a heterotetramer between two alpha and two beta subunits, where the alpha subunits mediate DNA binding through ETS domains, and the beta subunits mediate transcriptional activation [16]. For NRF-2α, several homologues are encoded by the fly genome: Delg, Ets21C, Pointed, Ets65A, Ets96B, Aop and Eip74EF (in decreasing order of homology). For NRF-2β, only one homologue has been found: CG32343. For three of these homologues, we induced homozygous mutant clones as described above and tested for mitochondrial defects. Two of them, *ets21C (ets21C^f03639^*) and *pointed* (*pnt^Δ88^*), did not show mitochondrial defects in the larval fat body (data not shown). In contrast, *delg* mutants did show such defects (see below), thus we focused on whether Delg could mediate the nutrient-specific regulation of mitochondria.

As published earlier [17], *Drosophila* Delg (CG6338; *Drosophila* Ets like gene) is a close homologue to mammalian NRF-2α, being 39% identical in amino acids sequence. In particular, the ETS domain is highly conserved (Fig. 2A), and 12 of the 13 residues that bind NRF-2β [18] are also conserved (data not shown). Delg was first identified as one of several *Drosophila* proteins containing an ETS domain [19], [20], [17]. Specific mutants have developmental defects, particularly during oogenesis [21], [22], [23]. A null mutant background (*delg^613^*/Df(3R)ro^80b^) is lethal during pupal stages, whereas a hypomorphic allele (*delg^tne^*) gives raise to viable but sterile adults [21]. To stain for mitochondria, we first used MitoTracker, which gave an abundant staining in the cytoplasm of wild type fat body cells. In contrast, *delg* null mutant cells showed a strong decrease in staining. Although reduced, mutant cells still retained staining that localized in a perinuclear manner (Fig. 2B). When quantified, we noted a 30% reduction in MitoTracker staining in *delg* mutant cells (Fig. 2C). Since these stainings were done on fixed tissues, they reflect mitochondrial abundance, but not mitochondrial activity. To further assay mitochondrial mass, we used NAO, which specifically labels the mitochondrial phospholipid cardiolipin, and is commonly used as a good readout to estimate mitochondrial mass. As seen in Fig. 2D, *delg* mutant cells showed a strong reduction in NAO. Again, residual mitochondria were concentrated around the nucleus. Finally, using electron microscopy, we noticed that *delg* mutants had similar numbers of mitochondria, but mitochondria were strongly reduced in size, being on average 50% smaller in area (Fig. 2E and 2F). To test whether this effect is cell-autonomous, we induced *delg* homozygous mutant clones using the Flp/FRT system. Mutant cells, recognized by the absence of GFP, showed a strong reduction in MitoTracker (Fig. 2G). Taken together, these data demonstrate that mitochondrial mass is reduced in *delg* mutant fat body cells in a cell-autonomous manner. In contrast, ectopic expression of Delg did not result in an increase in mitochondrial abundance (data not shown), demonstrating that Delg is required but not sufficient to control mitochondrial mass *in vivo*.

### Delg functions specifically in the larval fat body

To test whether the mitochondrial defects in the *delg* mutant are specific for the fat body, we stained for mitochondria in other larval tissues, including the gut, salivary gland, trachea and imaginal discs. Importantly, *delg* mutants did not show any changes in mitochondrial abundance or morphology in these tissues (Fig. S2A; Data not shown). Moreover, expression of exogenous Delg primarily in the fat body complemented the lethality of *delg* null alleles (see Materials and Methods). These data demonstrate that Delg functions primarily in the larval fat body, and is therefore a prime candidate to mediate the nutrient-specific control of mitochondria described above.

**Figure 3:**
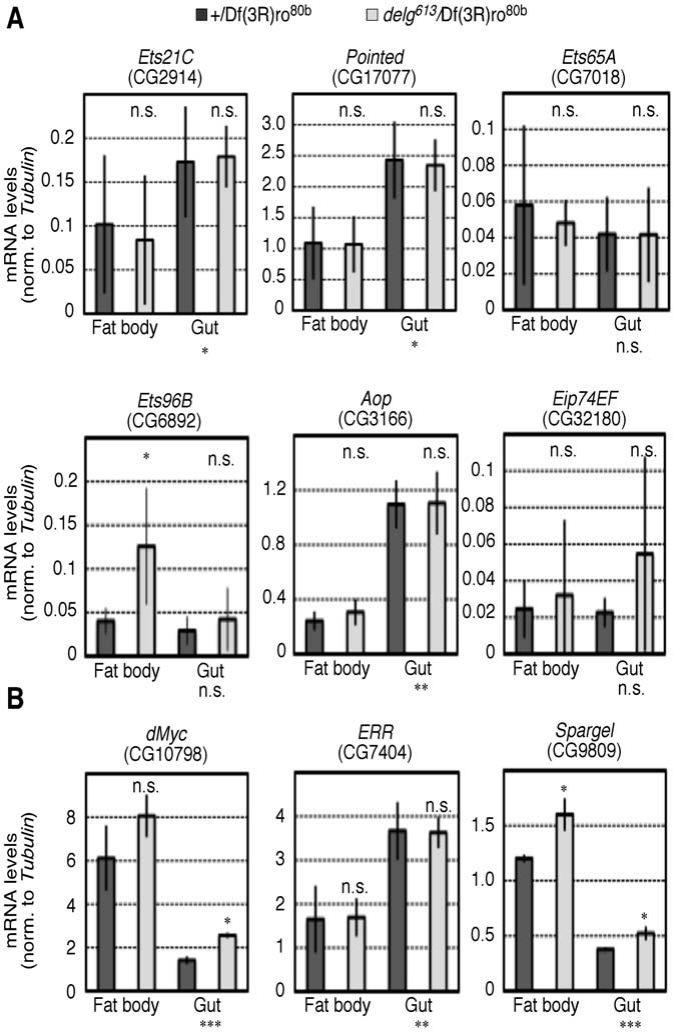
Expression levels in the fat body and the gut of candidate transcription factors to control the expression of genes encoding mitochondrial proteins. The larval fat body or gut was dissected from mid-third instar larvae (4d AED for +/Df(3R)ro^80b^, and 6d AED for *delg^613^*/Df(3R)ro^80b^), and mRNA levels were determined using qPCR and normalized to *Tubulin* levels. (A) *Drosophila* homologues to mammalian NRF-2α are shown. (B) *dMyc*, *Estrogen-related receptor* (*ERR*) and *Spargel* (*Drosophila* PGC-1 homologue) levels are shown. Significance between the fat body and the gut for control animals is shown below the charts, and significance between control and *delg* mutant is shown within the charts.

Even in a *delg* null mutant background, mitochondria are still present in the larval fat body. Moreover, Delg is expressed in the larval gut to similar levels as in the fat body (data not shown), yet we did not observe mitochondrial phenotypes in *delg* mutants (Fig. S2). Thus redundant factors, especially in the gut, must exist. To identify such factors, we dissected larval fat bodies and guts from mid-third instar animals, either control or *delg* mutant. qPCR was then used to measure mRNA levels of the *Drosophila* NRF-2α homologues mentioned above. Three of them, *Ets21C*, *Pointed* and *Aop*, were expressed at higher levels in the gut, compared to the fat body, thus potentially explaining the absence of phenotypes in *delg* mutant gut cells. Moreover, *Ets96B* was expressed at higher levels in the *delg* mutant fat body, compared to the control fat body (Fig. 3A). A detailed genetic characterization including double mutants between Delg and other NRF-2α homologues will be required to directly test for redundancy.

In mammalian systems, NRF-1 and NRF-2 function together with other transcription factors to drive the expression of genes encoding mitochondrial proteins. These factors include ERRα, c-Myc, SP1, YY1 as well as the transcriptional co-activators to the PGC-1 family [14], [4]. To test whether their fly homologues could function in a redundant manner, we measured mRNA of these genes in the larval fat body and the gut as done above. Whereas *dMyc* and *ERR* levels were not significantly elevated in *delg* mutant fat bodies, Spargel, the *Drosophila* PGC-1 homologue was expressed at higher levels. Moreover, *dMyc* and *Spargel* were also expressed at higher levels in the *delg* mutant gut, compared to control gut (Fig. 3B). As mentioned below, Delg and Spargel function in a redundant manner to control mitochondrial biogenesis in the larval fat body (Tiefenböck et al., *under revision*). Thus redundancy among multiple factors might be common for the expression of genes encoding mitochondrial proteins.

### Reduced expression levels of genes encoding mitochondrial proteins in *delg* mutants

**Figure 4:**
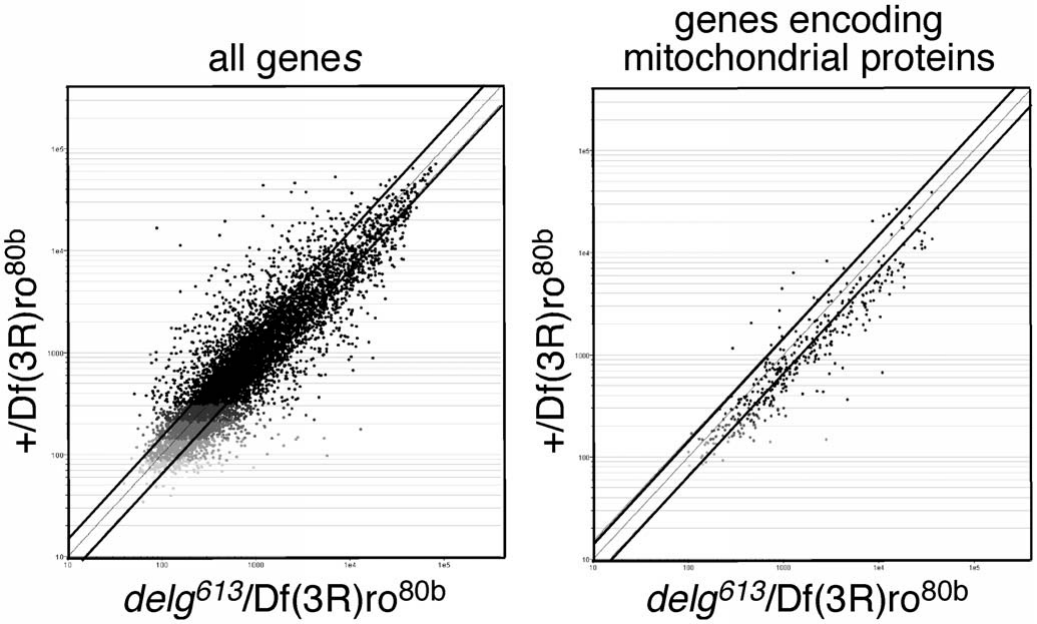
Reduced expression of genes encoding mitochondrial proteins in *delg* mutants. Schematic representation of microarray data using fat body mRNA dissected from late L3 larvae. Control: +/Df(3R)ro^80b^, mutant: *delg^613^*/Df(3R)ro^80b^. Left: All genes are shown. Right: Genes encoding mitochondrial proteins are shown. Diagonal lines indicate 1.5 fold up- or downregulation. Shown are averages of 3 biological replicates.

To test whether Delg is required for proper expression levels of genes encoding mitochondrial proteins, we performed microarray analysis using mRNA from dissected fat bodies. Indeed, 55% of annotated genes encoding mitochondrial proteins were at least 1.5fold downregulated in the *delg* mutant compared to control (Fig. 4 and Table S1). These genes are involved in respiration, energy transport, ATP synthesis and pyruvate metabolism. Besides these mitochondria-related functions, genes involved in other compartments were also affected, including ribosomes, the Golgi apparatus and the endoplasmatic reticulum. We have not addressed yet whether this reflects non-mitochondrial functions.

### Increased OXPHOS activity of individual mitochondria in *delg* mutant fat bodies

**Figure 5:**
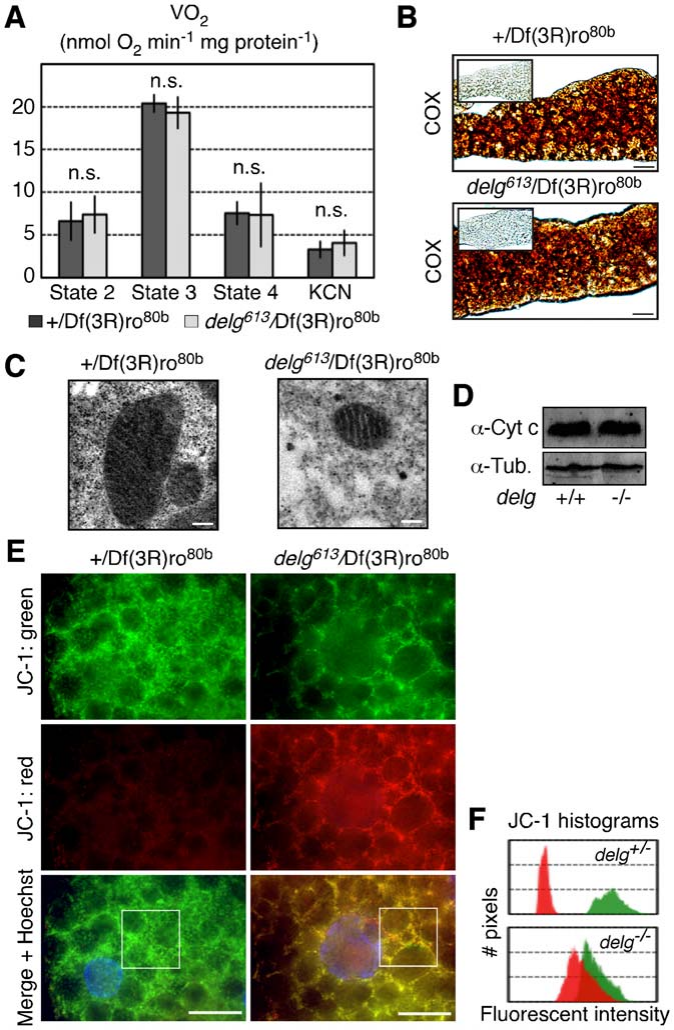
Normal mitochondrial activity in *delg* mutant fat bodies. (A) Oxygen consumption assays on digitonin-permeabilized dissected fat bodies as done in Fig. 1D. (B) Cytochrome c oxidase (COX) staining in larval fat bodies. The addition of COX-inhibitor KCN shows the specificity of the stain (shown as insets). (C) Electron microscopy images, showing normal cristae in *delg* mutants. (D) Western blot using larval fat body extracts, showing similar Cytochrome c levels in both genotypes. Anti Tubulin was used as a loading control. (E) JC-1 stainings in unfixed fat bodies. The predominant green signal in the heterozygous control indicates low mitochondrial membrane potential. The red signal in the *delg* mutant indicates high potential. White boxes outline the areas used for the histograms shown in F. (F) Quantification of JC-1 histograms, showing the intensity of red and green fluorescence. Bar equals 20 µm, except 0.2 µm in E.

To test whether the reduced expression of genes encoding mitochondrial proteins would affect respiration similarly to low-yeast food conditions described above, we repeated the respiration assays using dissected fat bodies from *delg* mutant larvae. Surprisingly, for none of the respiration states, we detected any difference compared to control backgrounds (Fig. 5A). Importantly, oxygen consumption in these assays was normalized to total fat body protein, and not to mitochondrial protein. Thus, since *delg* mutant tissue contains less mitochondrial mass, this indicates that the residual mitochondria must be more active to compensate for the reduced mass. To support this conclusion, we stained for Cytochrome c oxidase (COX) activity, reflecting complex IV of the mitochondrial electron transport chain. Indeed, *delg* mutant cells showed an identical COX activity compared to control (Fig. 5B). Moreover, individual mitochondria, although reduced in size, showed no morphological defects (Fig. 5C), and western blots demonstrated no change in Cytochrome c protein levels (Fig. 5D). These data are consistent with the model that individual mitochondria are more active, leading to a tissue with normal OXPHOS activity. These finding imply an increased inner-mitochondrial membrane potential in the *delg* mutant. We therefore used JC-1, a dye that fluoresces green under low inner-mitochondrial membrane potential, and red when the potential is high. When assayed in control fat bodies, green fluorescent was predominant, demonstrating low potential. In contrast, *delg* mutants showed an increased red fluorescence, indicating increased mitochondrial membrane potential (Fig. 5E and F). Taken together, although mitochondrial mass is reduced, remaining mitochondria show increased activity, leading to cells and tissues with normal OXPHOS activity. To our knowledge, this phenotype has not been described for any of the transcription factors involved in mitochondrial biogenesis. Our findings are very surprising given the reduced expression of OXPHOS genes in the *delg* mutant (Fig. 4). At least two models can explain this paradox: First, a transcription-independent mechanism exists to maintain normal protein or activity levels to cope with reduced expression in the *delg* mutant. Such a mechanism could affect either the translation of nuclear-encoded mitochondrial proteins, protein import into mitochondria, processing into functional complexes and/or the activation and turnover of these proteins. In *Drosophila*, these post-transcriptional regulations are very poorly understood, and biochemistry is required to address these questions. However, the *Drosophila* fat body yields only limiting amounts of material, and specific antibodies for fly proteins are currently not available. Thus this idea cannot be tested directly at present time. Second, factors that are rate limiting for OXPHOS activity are not affected by the Delg mutation. Indeed, we found normal mRNA and protein levels of Cytochrome c (data not shown and Fig. 5D). Using allelic series of *Cyt-c-p* hypomorphic mutants, as well as overexpression studies, we will test whether Cytochrome c is limiting for OXPHOS activity under physiological conditions.

### Delg does not mediate the reduction in respiration in response to nutrient starvation

**Figure 6:**
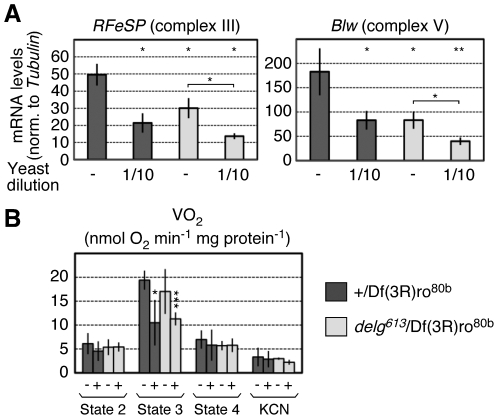
Delg does not mediate the nutrient-specific control of respiration activity. All animals were taken as mid-third instar larvae: control normal food: 4 days AED, control low yeast: 8 days AED, *delg* normal food 6 days AED and *delg* low yeast 10 days AED. Larvae were grown in normal or low yeast food as in Fig. 1. (A) qPCR for *RFeSP* and *Blw* mRNA levels, normalized to *Tubulin* levels. (B) Oxygen consumption assays on digitonin-permeabilized dissected fat bodies as done in Fig. 1D. ‘−’ indicates normal food, ‘+’ indicates low yeast food. In all cases, significance is indicated compared to normal fed control larvae, except in A, where in addition normal vs. low-yeast fed *delg* mutants are compared.

Given the different phenotypes between *delg* mutants and low-yeast food in respect to oxidative phosphorylation, it is unlikely that Delg mediates the reduction in respiration upon nutrient starvation. Still, we used qPCR to measure *RFeSP* and *Blw* levels in *delg* mutants, grown in normal or low yeast food. As expected from the microarray data, *delg* mutants showed a reduced expression of the two genes when mRNAs were isolated from dissected fat bodies. Importantly, when *delg* mutants were grown under low-yeast food, we detected additive decreases compared to *delg* mutants alone and control animals grown under low-yeast (Fig. 6A). This strongly suggests that factors other than Delg are involved to mediate the nutrient-dependent expression of these genes. To further characterize OXPHOS activity under these conditions, we repeated the respiration assays: Fat bodies from *delg* mutants, grown under low-yeast, showed a very similar drop in state 3 respiration when compared to control animals (Fig. 6B). These data demonstrate that Delg does not mediate the reduction in OXPHOS activity seen under low-yeast conditions.

### Delg is required for the control of mitochondrial abundance and glutamine metabolism in response to nutrient starvation

**Figure 7:**
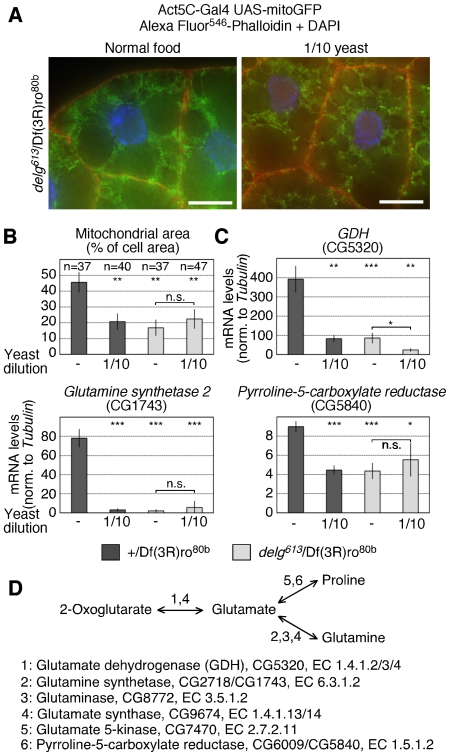
Delg mediates mitochondrial abundance and expression levels of enzymes involved in glutamine metabolism in response to nutrients. For all experiments, fat bodies were dissected from larvae grown as in Figure 6. (A) Alexa Fluor^546^-phalloidin staining of larval fat bodies, expressing UAS-mitoGFP. Shown are *delg* homozygous mutants; the corresponding controls are shown in Fig. 1A. Bar equals 20 µm. (B) Quantification of A. Mitochondrial area per cell was measured as in Fig. 1C. (C) Transcript levels for enzymes involved in glutamine metabolism were measured using qPCR, and normalized to *Tubulin* levels. Significance shown on top compares to normal fed controls, and significance between normal fed and low-yeast fed *delg* mutants is shown below. (D) Schematic representation of glutamine metabolism and the enzymes involved in 2-oxoglutarate, glutamate and proline synthesis.

To test whether Delg might mediate the reduction in mitochondrial abundance upon low-yeast starvation, we grew *delg* mutants under low yeast and labeled mitochondria using mitoGFP. Compared to low-yeast controls or normal-fed *delg* mutants, which both showed similar defects in mitochondrial abundance, we did not detect additive defects in mitochondrial abundance (Fig. 7A and B). We conclude that nutrients regulate mitochondrial abundance in a strictly Delg-dependent manner. Next, we measured transcript levels of the enzymes involved in glutamine metabolism. In normal-fed *delg* mutants, *GDH*, *Glutamine synthetase 2* and *Pyrroline-5-carboxylate reductase* levels were reduced to very similar levels compared to normal-fed controls. Importantly, with the exception of GDH, we did not detect additive defects when *delg* mutants were grown under low-yeast conditions (Fig. 7C). Taken together, Delg is critical to adjust to adjust mitochondrial abundance and the expression levels of genes encoding enzymes involved in glutamine metabolism to nutrient availability. This implies a model where Delg coordinates glutamine metabolism to mitochondrial mass.

### Cyclin D/Cdk4 require Delg to drive growth and mitochondrial biogenesis

**Figure 8:**
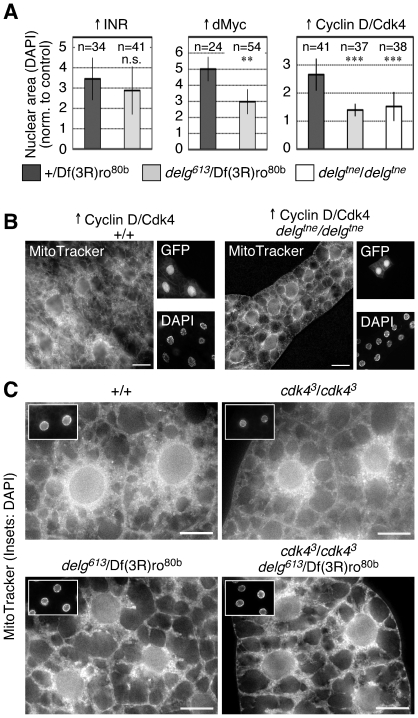
Mitochondrial biogenesis in response to Cyclin D/Cdk4. (A) UAS-INR (left), UAS-dMyc (middle) or UAS-Cyclin D/UAS-Cdk4 (right) were expressed in random clones in the fat body using the hs-Flp/Tubulin>CD2>Gal4 UAS system. Control background: dark grey; 4 days AED, *delg^613^*/Df(3R)ro^80b^ background: light grey; 6 days AED, *delg^tne^*/*delg^tne^* background: white; 6 days AED. Shown are nuclear sizes, based on DAPI stainings, normalized to internal controls (neighboring cells not expressing the growth drivers). (B) Cyclin D/Cdk4 was expressed in the indicated background in random clones marked by GFP as in A. MitoTracker stainings show that Cyclin D/Cdk4 is sufficient to stimulate mitochondrial biogenesis in a control, but not in a *delg* mutant background. (C) MitoTracker stainings from mid-third instar larvae (4 days AED for control and *cdk4*, 6 days AED for *delg* and *delg cdk4* double mutant). Both *cdk4* or *delg* single mutants have reduced mitochondrial abundance. No additive effect is seen in the *cdk4 delg* double mutant. Note that control and *delg* single mutant pictures are identical to the ones shown in Fig. 2B. Bar equals 20 µm.

So far, we demonstrated that Delg mediates a critical link between nutrients and mitochondria. We therefore hypothesized that factors that drive cellular growth in a nutrient-dependent manner could function through Delg. In the larval fat body, only few such factors have been characterized, including the insulin/TOR pathway [24], the transcription factor dMyc [25], and the cyclin-dependent protein kinase complex Cyclin D/Cdk4 [26], [27]. To test whether Delg is functionally linked to any of these pathways, we used gain-of-function to induce overgrowth in clones in a wild type or a *delg* mutant background. Since endoreplication correlates with cell size and thus with cellular growth, we used DAPI stainings to measure overgrowth. As expected, expression of the insulin receptor (INR) was sufficient to stimulate growth. Importantly, this was not significantly changed in *delg* mutants, demonstrating that Delg is not required for INR to stimulate growth (Fig. 8A, left). When dMyc was expressed, the growth-promoting function was partially suppressed (Fig. 8A, middle). Since mammalian Myc is required for mitochondrial biogenesis [28], these data suggest a possible analogues function in *Drosophila*. However, we have shown previously that dMyc overexpression does not drive mitochondrial abundance in *Drosophila*, and that overgrowth driven by dMyc did not depend on mitochondrial activity in the larval fat body [29]. Therefore, we did not further investigate a possible functional link between dMyc and Delg. In contrast, Cyclin D/Cdk4 did require Delg to stimulate overgrowth: Whereas in a control background, ectopic expression of Cyclin D/Cdk4 led to a 2–3 fold increase in nuclear area, this was completely abolished in a *delg* null or hypomorphic mutant background (Fig. 8A, right). Furthermore, using phalloidin stainings to assay cell size, we noted that ectopic expression of Cyclin D/Cdk4 led to larger cells, which again was abolished in the *delg* mutants (data not shown), demonstrating a functional link between Cyclin D/Cdk4 and Delg.

We have shown previously that ectopic expression of Cyclin D/Cdk4 leads to increased mitochondrial abundance, and increased mitochondrial activity [29]. To test whether Delg is functionally required for Cyclin D/Cdk4's effect on mitochondria, we again used MitoTracker stainings in the larval fat body. As shown in Fig. 8B, ectopic expression of Cyclin D/Cdk4, marked by co-expression of GFP, led to increased stainings, where most of the cytoplasm was filled with mitochondria. This increase was completely abolished in the *delg* mutant background (Fig. 8B). Moreover, overexpression of Cyclin D/Cdk4 led to a strong increase in mitochondrial COX activity [29], which was also suppressed by in the *delg* homozygous mutant background (data not shown). This demonstrates that Delg mediates a link between Cyclin D/Cdk4 and mitochondrial abundance and activity.

### Reduced mitochondrial abundance in *cdk4* mutants

To further characterize Cyclin D/Cdk4's effect on mitochondria, we used previously characterized mutants. Flies lacking Cdk4 are viable, but show reduced growth levels [26]. Using qPCR, we measured mRNA levels from dissected fat bodies of the genes involved in glutamine metabolism. *Glutamate synthase* showed a significant, 3.5-fold decrease in a *cdk4* null mutant background, compared to control (data not shown), suggesting that glutamine metabolism can be affected in response to Cdk4 function. When mitochondria were stained in the larval fat body using MitoTracker, *cdk4* mutants showed reduced mitochondrial abundance, but to a lesser degree than *delg* mutants (Fig. 8C). Thus whereas Cyclin D/Cdk4 gain-of-function is sufficient to stimulate mitochondrial abundance, mutants have reduced mitochondrial mass, at least in the larval fat body. Importantly, the *cdk4 delg* double mutant did not show an additive mitochondrial defect compared to the *delg* single mutant (Fig. 8C). We conclude that Cyclin D/Cdk4 functions through Delg to target mitochondria.

## Discussion

In *Drosophila* as well as other insects, the fat body is the major organ for de novo biosynthesis of fatty acids, leading to the storage of lipids as triacylglycerols. Equally important, the fat body is known to release amino acids, such a glutamine and proline [30], which are synthesized from the mitochondrial metabolite 2-oxoglutarate. Therefore, one would expect that mitochondrial mass and activity are regulated in response to nutrients in the larval fat body. Indeed, we show strong decreases in mitochondrial abundance, respiration activity as well as expression levels of enzymes involved in glutamine and proline metabolism under low-yeast food. Under these feeding conditions, amino acids and fatty acids, which are both provided by yeast, become limited. *Delg* mutants show very similar phenotypes compared to normal fed controls, and do not show additive phenotypes in respect to mitochondrial abundance and amino acid metabolism upon low-yeast food. We therefore propose that Delg functions as a transcription factor to coordinate mitochondrial functions according to nutrient availability. One of these aspects is to adjust the synthesis of non-essential amino acids to the uptake of essential amino acids. In this respect, de novo synthesis of L-glutamine is particularly interesting, as the efflux of its cytoplasmic pool is used, both in mammals and *Drosophila*, for import of essential amino acids [2]. Our model proposes that Delg either directly senses nutrients, most likely amino acids, or is controlled by upstream sensors. Since the nutrient-sensitive Cyclin D/Cdk4 pathway functions through Delg, the latter seems more likely. Given the key role of the fat body in metabolic homeostasis of the whole animal, one might expect that fat body mitochondria be regulated differently from mitochondria in other tissues. Indeed, our phenotypes were specific to the fat body, demonstrating that Delg functions primarily in this tissue to coordinate the different anabolic and catabolic functions of mitochondria.

Mammalian NRF-2 was identified through its binding to the promoters of cytochrome c oxidase (COX) subunits [31], [32], and previously had been purified as GABP [33]. Active NRF-2 is a heterotetramer consisting of two alpha and two beta subunits. The alpha subunits mediate DNA binding, which requires direct GGAA/T repeats in the promoters. Accordingly, electromobility assays as well as luciferase reporter assays have shown that these motifs are functionally important. This is particular well understood for genes encoding electron transport proteins [34], [35], [31], [36], [37], [38], as well as for mitochondrial protein import [39], [40], [41]. Furthermore, direct NRF-2 binding to several promoters was shown by chromatin immunoprecipitation [42], [38]. Thus biochemical evidence links NRF-2/GABP to the transcriptional control of nuclear genes encoding mitochondrial proteins. Accordingly, RNAi studies found reduced expression of several COX subunits in cells having reduced NRF-2α levels, leading to reduced COX activity [43]. Surprisingly, genetic data have not supported the biochemical data: MEFs lacking NRF-2α/GABPα do not have reduced mRNA or protein levels of several putative NRF-2 targets, and mitochondrial phenotypes were not reported [44]. *Drosophila* Delg is the closest fly homologue to mammalian NRF-2α. Two-hybrid data show that Delg can bind to the *Drosophila* NRF-2β homologue CG32343 [45], and preliminary data show that *CG32343* mutants have mitochondrial defects very similar to *delg* mutants (data not shown). Taken together, these data show that Delg functions analogues to mammalian NRF-2α, and our data are the first genetic evidence that links any member of the NRF-2α family to mitochondrial biogenesis.

Of particular interest is the strong reduction in mitochondrial size in the *delg* mutants (Fig. 2E and 2F). This implies that mitochondrial fusion might be defective, and/or that fission occurs at an increased rate. Indeed, based on our microarray data, expression of *Opa1-like* (CG8479), the fly homologue to mammalian fusion protein OPA1 [1], showed a significant, 2-fold reduction in expression in *delg* mutant fat body samples (data not shown). In contrast, fly homologues to mammalian Mitofusins, which are well-established fusion factors (fly homologues *Marf*/*CG3869* and *Fuzzy onions*/*CG4568*), were not differently expressed (data not shown). More work is required to test whether *delg* mutant have defects in mitochondrial fusion. In addition, when *delg* homozygous mutant clones were induced, we noted a strong reduction in cell size, yet the nuclear size, shown by the DAPI staining, was not changed (Fig. 2G), demonstrating growth defects, This is surprising, since endoreplication, and thus nuclear size, normally correlates with cell size in this tissue. Since mitochondrial biogenesis has been shown to correlate with nuclear DNA synthesis [46], [47], our data suggest that Delg might be involved to link S-phase and potentially cell size to mitochondrial mass. Future work will be required to address this hypothesis.

Genes involved in mitochondrial OXPHOS activity, including *RFeSP* and *Blw*, showed similar reduced expression in the *delg* mutant or under low yeast nutrition. Importantly, we detected additive defects when *delg* mutant were grown under low yeast (Fig. 6A). Moreover, when oxygen consumption was measured in permeabilized fat body tissues, state 3 respirations were strongly affected by low yeast nutrition, yet this was independent of Delg (Fig. 6B). This demonstrates that factors other than Delg must regulate mitochondrial OXPHOS activity in response to nutrients. One candidate is Spargel/CG9809, the fly homologue to mammalian PGC-1 proteins [9], which are transcriptional coactivators that control mitochondrial mass and activity in response to external stimuli [4]. Indeed, we found that Spargel functions in parallel to Delg, and mediates a link between insulin-signalling and the expression of genes encoding mitochondrial proteins (Tiefenböck et al., *under revision*). Therefore, Delg and Spargel mediate two parallel pathways that control mitochondrial mass and OXPHOS activity in response to nutrients.

*Drosophila* Cyclin D/Cdk4 is a cyclin-dependent protein kinase complex, and controls cellular growth levels in addition to regulating cell cycle progression [26], [27], [48]. Importantly for this study, overgrowth induced by ectopic expression of Cyclin D/Cdk4 is insensitive to nutrient conditions [49], demonstrating that the Cyclin D/Cdk4 pathway is nutrient-responsive. We now show that ectopic expression of Cyclin D/Cdk4 in the larval fat body is sufficient to drive mitochondrial abundance in a Delg-dependent manner (Fig. 8B), suggesting a mechanism where Cyclin D/Cdk4 coordinate growth levels and mitochondrial mass. Furthermore, one would expect that the transcriptional activity associated with Delg is regulated in response to Cyclin D/Cdk4. Indeed, using lacZ insertions into the loci of several genes encoding mitochondrial proteins, including *Blw*, clonal expression of Cyclin D/Cdk4 was sufficient to stimulate expression of these genes. However, this effect was restricted to wandering third instar larvae, but was not seen in feeding L1, L2 or L3 larvae (data not shown). Moreover, when larvae were grown under low-yeast conditions, ectopic expression of Cyclin D/Cdk4 led to reduced expression of these genes, again based on lacZ insertions (data not shown). We conclude that Cyclin D/Cdk4 is not a general activator of Delg function, but might mediate a nutrient and/or developmental-dependent control.

In mammals, D-type cyclins bound to Cdk4 or Cdk6 are best characterized for their functions during cell cycle progression, but several reports have shown additional roles, in particular the regulation of multiple transcription factors by direct binding [50]. Importantly for this study, mitochondrial size and activity are regulated in response to mammalian Cyclin D1: Knockdown or knockout of Cyclin D1 leads to larger mitochondria that are more active [51], [52]. Conversely, ectopic expression of Cyclin D1 inhibits mitochondrial activity, a function that requires binding to Cdk4 [52]. Moreover, Cyclin D1 and NRF-1 are functional linked: Cyclin D1 stimulates NRF-1-dependent transcription, the two proteins interact, and a protein kinase associated to immunoprecipitated Cyclin D1 can phosphorylate NRF-1 [52]. This suggests a mechanism where Cyclin D1/Cdk4 inhibit mitochondrial mass and activity through inhibition of NRF-1 function. Thus D-type cyclins bound to Cdk4 have opposite effects on mitochondrial mass in mammals compared to flies, suggesting that different mechanisms have been adopted during evolution to control mitochondria.

## Materials and Methods

### Fly stocks

Following fly lines were used: *delg^613^* and *delg^tne^* [21], UAS-mitoGFP [53], UAS-Cyclin D, UAS-Cdk4 [27], *cycd^1^* [48], *cdk4^3^* [26], hsFlp^122^ Act>CD2>Gal4 UAS-GFP [54], hsFlp^122^ Tubulin>CD2>Gal4 UAS-GFP [55], UAS-INR [56], UAS-dMyc [57]. Df(3R)ro^80b^, FRT82-GFP, Act5C-Gal4 are all from Bloomington Stock Center (Indiana University, USA). For rescue experiments, the Delg coding sequence was cloned into the pTWH vector (HA-tagged; *Drosophila* Gateway Collection, Carnegie Institution of Washington, USA), and transgenic files were obtained using standard procedures. UAS-HA-Delg rescues the lethality of *delg^613^*/Df(3R)ro^80b^ when expressed using hs-Gal4 or CS2-Gal4 (kindly provided by M. Pankratz). CS2-Gal4 is derived from the *ppl* promoter, and is expressed strongly in the larval fat body and weakly in salivary glands, tracheae, gastric cacae and the epidermis.

### Low-yeast fly food

1l of normal food: 100 g yeast, 75 g sugar, 55 g corn, 10 g flour, and 8 g agar. For low yeast food, only 10 g yeast was added. The mixture was boiled, cooled to ∼60°C, and 0.5 g nipagin and 1 g nipasol were added, both dissolved in ethanol. Animals grown on low yeast food are viable and fertile, and show on average a 30% reduction in adult wet weight (data not shown).

### Biochemistry

Quantitative Western Blots were done using the Odyssey Infrared Imagines System (LI-COR Biosciences) using Alexa Fluor^680^ secondary antibodies (Molecular Probes). Anti-Cytochrome c (BD Biosciences) was used at 1/400 and anti Tubulin (Sigma) at 1/4000.

### Microscopy

MitoTracker and COX stainings were preformed as [29]. JC-1 (5 µM; Molecular Probes) or NAO (1 µM; Sigma) were added to unfixed, inverted larvae in PBS, stained for 15 min in the dark, fat bodies were mounted in 80% glycerol and samples were imaged within 2 h. Hoechst^33342^ (Molecular Probes) was used at 0.5 µg/ml. Alexa Fluor^546^-phalloidin (Invitrogen) was added after fixation to inverted larvae at 0.8 U/200 µl in PBS + 0.02% Triton X-100, stained 60 min, washed in PBS/Triton and mounted. Images were acquired on a Deltavision Olympus K70 microscope using a CoolSNAP HQ camera (Photometrics). Serial Z-sections were acquired at 0.2 µm distance and deconvolved using Softworx software (Applied Precision). Shown are projections of three subsequent sections. For electron microscopy, samples were prepared as described in [58], and images were acquired on a FEI Morgagni 268 microscope.

### Respiration

Respiration assays were performed as described in [13]. In brief, fat bodies from 40 larvae were dissected, permeabilized with digitonin (25 µM), and O_2_ concentration was measured using a Clark electrode after serial addition of pyruvate and proline (each at 10 mM; state 2), ADP (2 mM; state 3), and atractyloside (50 µM; state 4). KCN (0.5 mM) was added at the end to measure mitochondrial-independent respiration. O_2_ consumption was normalized to total fat body protein. All chemical were from Sigma.

### Microarray

Fat bodies were dissected and put into RNA later (Roche). To reduce the fat amount, the tissues were lysed in Qiazol lysis buffer (Roche) and a Chloroform extraction was performed. NucleoSpin RNA II (Macherey-Nagel) was used for RNA isolation. Samples were analyzed by the Functional Genomics Center Zurich (FGCZ; http://www.fgcz.ethz.ch) using the Affymetrix GeneChip *Drosophila* Genome 2.0 Array. For the evaluation GeneSpring GX Software (Agilent Technologies) was used. Original data have been deposited at NCBI's Gene Expression Omnibus [59], are MIAME compatible and can be obtained using the series accession number GSE14058.

### Quantitative PCR

For mRNA levels, dissected tissues were put into RNA later (Roche). RNA was isolated using NucleoSpin RNA II (Macherey-Nagel). For cDNA synthesis, Ready-To-Go You-Prime First-Strand beads (GE healthcare) were used. Quantitative real time PCR was performed with the Light Cycler 480 (Roche). Primer sequences can be obtained upon request. In all cases, averages and standard deviations are shown from at least three biological replicates.

### Statistical analysis

In all experiments, significance was determined using the Student's t-distribution (two-tailed; two-sample equal variances). *** equals *P*<0.001; ** equals *P*<0.01; * equals *P*<0.05; ns: not significant.

## Supporting Information

Figure S1 (A) Colocalization of mitoGFP and MitoTracker. UAS-mitoGFP was expressed using Act5C-Gal4, and counterstained using MitoTracker. Shown is a single fat body cell from a wild type larva 4 days after egg deposition. Bar equals 20 µm. (B) Gene expression levels from dissected fat bodies. In all cases, mid-third instar larvae were taken: control normal food: 4 days AED, control low yeast: 8 days AED, delg normal food 6 days AED and delg low yeast 10 days AED. mRNA levels of genes encoding enzymes involved in glutamine metabolism were measured using qPCR, and normalized to Tubulin levels. Significance is indicated: n.s.: no statistical significance, one star: P<0.05, two stars: P<0.01, three stars: P<0.001 (Student's TTEST). Significance compared to normal fed control is shown on top, and significance between delg mutants (normal fed vs. 10% yeast food) is shown below. (C) Schematic representation of enzymes involved in glutamine metabolism.
(3.18 MB TIF)

Figure S2 No defects in mitochondrial morphology and abundance for delg mutants in non-fat body tissues. Anti-cytochrome c antibody stainings (BD Biosciences at 1/400 dilution) in larvae expressing Act5C-Gal4 UAS-mitoGFP, 4 days (delg613/+) or 6 days after egg deposition (delg613/Df(3R)ro80b). For the gut, pictures were taken close to the proventiculus. For salivary glands, pictures were taken close to the distal tip. For tracheae, only the GFP channel is shown, and images was taken from the dorsal trunk close to the posterior spiracles. Bar equals 20 µm.
(4.30 MB TIF)

Table S1 Microarray data show reduced expression of ∼55% of all genes encoding mitochondrial proteins. Fat bodies from wandering heterozygous control animals (+/Df(3R)ro80b) and delg mutants (delg613/Df(3R)ro80b) were dissected, and mRNA levels of all annotated genes were analyzed using the microarray technique (see Materials and Methods). Mitochondrial proteins were clustered as in (Sardiello et al. 2003). Shown are expression levels in the delg mutant, normalized to the expression in control animals. Shown are log2 values of three biological replicates. N/A: Not detected. Reference Sardiello, M., Licciulli, F., Catalano, D., Attimonelli, M., and Caggese, C. 2003. MitoDrome: a database of Drosophila melanogaster nuclear genes encoding proteins targeted to the mitochondrion. Nucleic Acids Res 31(1): 322–324.
(0.34 MB DOC)

## References

[1] Chan DC (2006). Mitochondria: dynamic organelles in disease, aging, and development.. Cell.

[2] Nicklin P, Bergman P, Zhang B, Triantafellow E, Wang H (2009). Bidirectional transport of amino acids regulates mTOR and autophagy.. Cell.

[3] DeBerardinis RJ, Lum JJ, Hatzivassiliou G, Thompson CB (2008). The biology of cancer: metabolic reprogramming fuels cell growth and proliferation.. Cell Metab.

[4] Scarpulla RC (2008). Transcriptional paradigms in mammalian mitochondrial biogenesis and function.. Physiol Rev.

[5] White KP, Rifkin SA, Hurban P, Hogness DS (1999). Microarray analysis of Drosophila development during metamorphosis.. Science.

[6] Arbeitman MN, Furlong EE, Imam F, Johnson E, Null BH (2002). Gene expression during the life cycle of Drosophila melanogaster.. Science.

[7] Zinke I, Schutz CS, Katzenberger JD, Bauer M, Pankratz MJ (2002). Nutrient control of gene expression in Drosophila: microarray analysis of starvation and sugar-dependent response.. EMBO J.

[8] Teleman AA, Hietakangas V, Sayadian AC, Cohen SM (2008). Nutritional control of protein biosynthetic capacity by insulin via Myc in Drosophila.. Cell Metab.

[9] Gershman B, Puig O, Hang L, Peitzsch RM, Tatar M (2007). High-resolution dynamics of the transcriptional response to nutrition in Drosophila: a key role for dFOXO.. Physiol Genomics.

[10] Puig O, Tjian R (2006). Nutrient availability and growth: regulation of insulin signaling by dFOXO/FOXO1.. Cell Cycle.

[11] Baker KD, Thummel CS (2007). Diabetic larvae and obese flies-emerging studies of metabolism in Drosophila.. Cell Metab.

[12] Colombani J, Raisin S, Pantalacci S, Radimerski T, Montagne J (2003). A nutrient sensor mechanism controls Drosophila growth.. Cell.

[13] Kuznetsov AV, Veksler V, Gellerich FN, Saks V, Margreiter R (2008). Analysis of mitochondrial function in situ in permeabilized muscle fibers, tissues and cells.. Nat Protoc.

[14] Kelly DP, Scarpulla RC (2004). Transcriptional regulatory circuits controlling mitochondrial biogenesis and function.. Genes Dev.

[15] Koushika SP, Soller M, DeSimone SM, Daub DM, White K (1999). Differential and inefficient splicing of a broadly expressed Drosophila erect wing transcript results in tissue-specific enrichment of the vital EWG protein isoform.. Mol Cell Biol.

[16] Rosmarin AG, Resendes KK, Yang Z, McMillan JN, Fleming SL (2004). GA-binding protein transcription factor: a review of GABP as an integrator of intracellular signaling and protein-protein interactions.. Blood Cells Mol Dis.

[17] The SM, Xie X, Smyth F, Papas TS, Watson DK (1992). Molecular characterization and structural organization of D-elg, an ets proto-oncogene-related gene of Drosophila.. Oncogene.

[18] Batchelor AH, Piper DE, de la Brousse FC, McKnight SL, Wolberger C (1998). The structure of GABPalpha/beta: an ETS domain- ankyrin repeat heterodimer bound to DNA.. Science.

[19] Pribyl LJ, Watson DK, Schulz RA, Papas TS (1991). D-elg, a member of the Drosophila ets gene family: sequence, expression and evolutionary comparison.. Oncogene.

[20] Chen T, Bunting M, Karim FD, Thummel CS (1992). Isolation and characterization of five Drosophila genes that encode an ets-related DNA binding domain.. Dev Biol.

[21] Schulz RA, Hogue DA, The SM (1993). Characterization of lethal alleles of D-elg, an ets proto-oncogene related gene with multiple functions in Drosophila development.. Oncogene.

[22] Schulz RA, The SM, Hogue DA, Galewsky S, Guo Q (1993). Ets oncogene-related gene Elg functions in Drosophila oogenesis.. Proc Natl Acad Sci U S A.

[23] Gajewski KM, Schulz RA (1995). Requirement of the ETS domain transcription factor D-ELG for egg chamber patterning and development during Drosophila oogenesis.. Oncogene.

[24] Grewal SS (2008). Insulin/TOR signaling in growth and homeostasis: A view from the fly world.. Int J Biochem Cell Biol.

[25] de la Cova C, Johnston LA (2006). Myc in model organisms: a view from the flyroom.. Semin Cancer Biol.

[26] Meyer CA, Jacobs HW, Datar SA, Du W, Edgar BA (2000). Drosophila Cdk4 is required for normal growth and is dispensable for cell cycle progression.. Embo J.

[27] Datar SA, Jacobs HW, de la Cruz AF, Lehner CF, Edgar BA (2000). The Drosophila cyclin D-Cdk4 complex promotes cellular growth.. Embo J.

[28] Li F, Wang Y, Zeller KI, Potter JJ, Wonsey DR (2005). Myc stimulates nuclearly encoded mitochondrial genes and mitochondrial biogenesis.. Mol Cell Biol.

[29] Frei C, Galloni M, Hafen E, Edgar BA (2005). The Drosophila mitochondrial ribosomal protein mRpL12 is required for Cyclin D/Cdk4-driven growth.. Embo J.

[30] Candy DJ, Becker A, Wegener G (1997). Coordination and integration of metabolism in insect flight. Comp. Biochem.. Physiol.

[31] Virbasius JV, Virbasius CA, Scarpulla RC (1993). Identity of GABP with NRF-2, a multisubunit activator of cytochrome oxidase expression, reveals a cellular role for an ETS domain activator of viral promoters.. Genes Dev.

[32] Gugneja S, Virbasius JV, Scarpulla RC (1995). Four structurally distinct, non-DNA-binding subunits of human nuclear respiratory factor 2 share a conserved transcriptional activation domain.. Mol Cell Biol.

[33] LaMarco KL, McKnight SL (1989). Purification of a set of cellular polypeptides that bind to the purine-rich cis-regulatory element of herpes simplex virus immediate early genes.. Genes Dev.

[34] Virbasius JV, Scarpulla RC (1991). Transcriptional activation through ETS domain binding sites in the cytochrome c oxidase subunit IV gene.. Mol Cell Biol.

[35] Carter RS, Bhat NK, Basu A, Avadhani NG (1992). The basal promoter elements of murine cytochrome c oxidase subunit IV gene consist of tandemly duplicated ets motifs that bind to GABP-related transcription factors.. J Biol Chem.

[36] Villena JA, Vinas O, Mampel T, Iglesias R, Giralt M (1998). Regulation of mitochondrial biogenesis in brown adipose tissue: nuclear respiratory factor-2/GA-binding protein is responsible for the transcriptional regulation of the gene for the mitochondrial ATP synthase beta subunit.. Biochem J.

[37] Ongwijitwat S, Wong-Riley MT (2004). Functional analysis of the rat cytochrome c oxidase subunit 6A1 promoter in primary neurons.. Gene.

[38] Ongwijitwat S, Wong-Riley MT (2005). Is nuclear respiratory factor 2 a master transcriptional coordinator for all ten nuclear-encoded cytochrome c oxidase subunits in neurons?. Gene.

[39] Blesa JR, Hernandez JM, Hernandez-Yago J (2004). NRF-2 transcription factor is essential in promoting human Tomm70 gene expression.. Mitochondrion.

[40] Blesa JR, Hernandez-Yago J (2006). Distinct functional contributions of 2 GABP-NRF-2 recognition sites within the context of the human TOMM70 promoter.. Biochem Cell Biol.

[41] Blesa JR, Prieto-Ruiz JA, Hernandez JM, Hernandez-Yago J (2007). NRF-2 transcription factor is required for human TOMM20 gene expression.. Gene.

[42] Gleyzer N, Vercauteren K, Scarpulla RC (2005). Control of mitochondrial transcription specificity factors (TFB1M and TFB2M) by nuclear respiratory factors (NRF-1 and NRF-2) and PGC-1 family coactivators.. Mol Cell Biol.

[43] Ongwijitwat S, Liang HL, Graboyes EM, Wong-Riley MT (2006). Nuclear respiratory factor 2 senses changing cellular energy demands and its silencing down-regulates cytochrome oxidase and other target gene mRNAs.. Gene.

[44] Yang ZF, Mott S, Rosmarin AG (2007). The Ets transcription factor GABP is required for cell-cycle progression.. Nat Cell Biol.

[45] Giot L, Bader JS, Brouwer C, Chaudhuri A, Kuang B (2003). A protein interaction map of Drosophila melanogaster.. Science.

[46] Martinez-Diez M, Santamaria G, Ortega AD, Cuezva JM (2006). Biogenesis and dynamics of mitochondria during the cell cycle: significance of 3′UTRs.. PLoS One.

[47] Schieke SM, McCoy JP,, Finkel T (2008). Coordination of mitochondrial bioenergetics with G1 phase cell cycle progression.. Cell Cycle.

[48] Emmerich J, Meyer CA, de la Cruz AF, Edgar BA, Lehner CF (2004). Cyclin d does not provide essential cdk4-independent functions in Drosophila.. Genetics.

[49] Datar SA, Galloni M, de la Cruz A, Marti M, Edgar BA (2006). Mammalian cyclin D1/Cdk4 complexes induce cell growth in Drosophila.. Cell Cycle.

[50] Coqueret O (2002). Linking cyclins to transcriptional control.. Gene.

[51] Sakamaki T, Casimiro MC, Ju X, Quong AA, Katiyar S (2006). Cyclin D1 determines mitochondrial function in vivo.. Mol Cell Biol.

[52] Wang C, Li Z, Lu Y, Du R, Katiyar S (2006). Cyclin D1 repression of nuclear respiratory factor 1 integrates nuclear DNA synthesis and mitochondrial function.. Proc Natl Acad Sci U S A.

[53] Guo X, Macleod GT, Wellington A, Hu F, Panchumarthi S (2005). The GTPase dMiro is required for axonal transport of mitochondria to Drosophila synapses.. Neuron.

[54] Neufeld TP, de la Cruz AF, Johnston LA, Edgar BA (1998). Coordination of growth and cell division in the Drosophila wing.. Cell.

[55] Scott RC, Schuldiner O, Neufeld TP (2004). Role and regulation of starvation-induced autophagy in the Drosophila fat body.. Dev Cell.

[56] Brogiolo W, Stocker H, Ikeya T, Rintelen F, Fernandez R (2001). An evolutionarily conserved function of the Drosophila insulin receptor and insulin-like peptides in growth control.. Curr Biol.

[57] Schreiber-Agus N, Stein D, Chen K, Goltz JS, Stevens L (1997). Drosophila Myc is oncogenic in mammalian cells and plays a role in the diminutive phenotype.. Proc Natl Acad Sci U S A.

[58] Rusten TE, Lindmo K, Juhasz G, Sass M, Seglen PO (2004). Programmed autophagy in the Drosophila fat body is induced by ecdysone through regulation of the PI3K pathway.. Dev Cell.

[59] Edgar R, Domrachev M, Lash AE (2002). Gene Expression Omnibus: NCBI gene expression and hybridization array data repository.. Nucleic Acids Res.

