# Supplementary figures and images for: Nutrition Controls Mitochondrial Biogenesis in the Drosophila Adipose Tissue through Delg and Cyclin D/Cdk4
Source: PLoS One. 2009 Sep 9;4(9):e6935. doi: 10.1371/journal.pone.0006935 (PMC2735006; doi:10.1371/journal.pone.0006935)

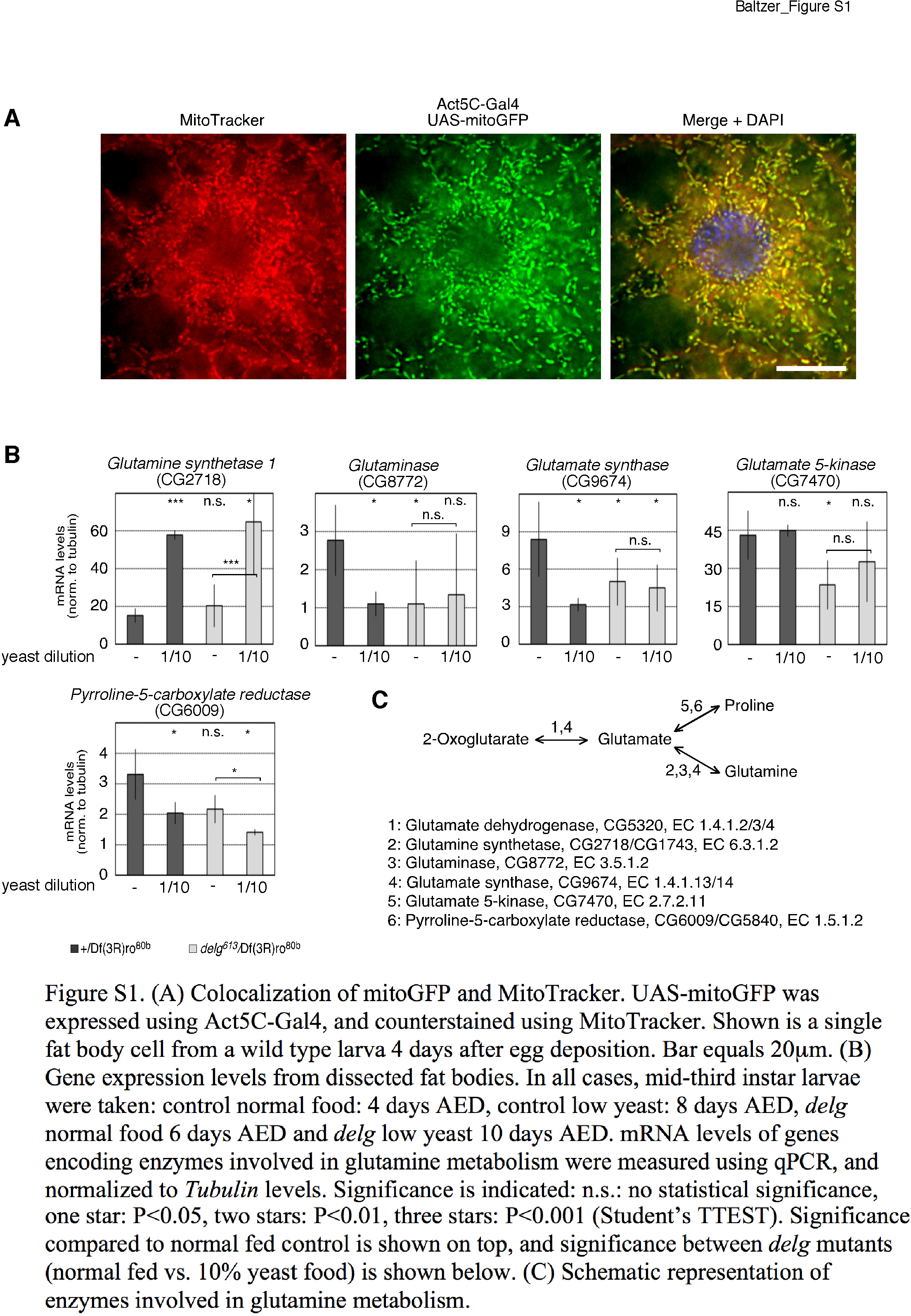

Supplement: Figure S1 — (A) Colocalization of mitoGFP and MitoTracker. UAS-mitoGFP was expressed using Act5C-Gal4, and counterstained using MitoTracker. Shown is a single fat body cell from a wild type larva 4 days after egg deposition. Bar equals 20 µm. (B) Gene expression levels from dissected fat bodies. In all cases, mid-third instar larvae were taken: control normal food: 4 days AED, control low yeast: 8 days AED, delg normal food 6 days AED and delg low yeast 10 days AED. mRNA levels of genes encoding enzymes involved in glutamine metabolism were measured using qPCR, and normalized to Tubulin levels. Significance is indicated: n.s.: no statistical significance, one star: P<0.05, two stars: P<0.01, three stars: P<0.001 (Student's TTEST). Significance compared to normal fed control is shown on top, and significance between delg mutants (normal fed vs. 10% yeast food) is shown below. (C) Schematic representation of enzymes involved in glutamine metabolism. (3.18 MB TIF) [file pone.0006935.s001.tif]

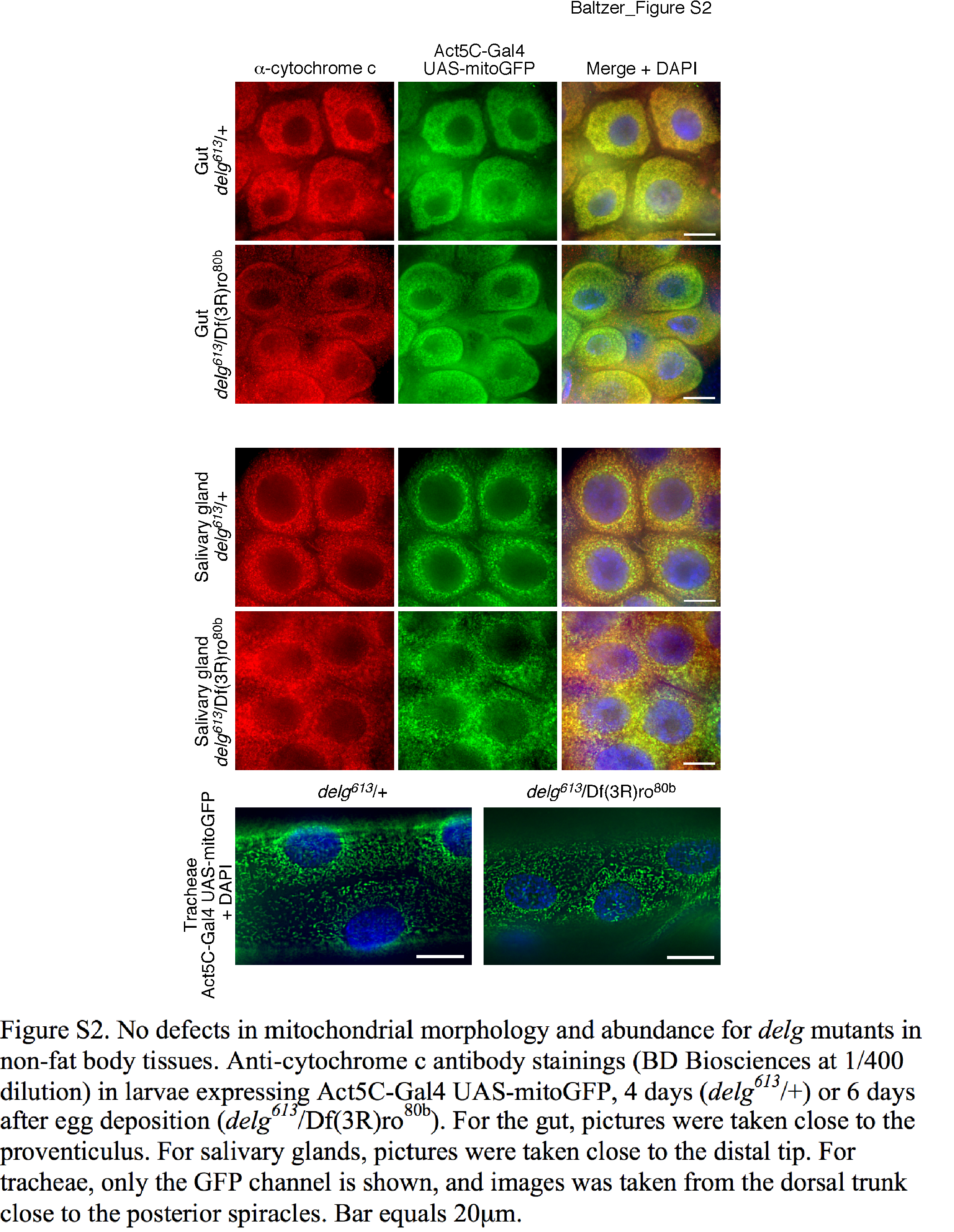

Supplement: Figure S2 — No defects in mitochondrial morphology and abundance for delg mutants in non-fat body tissues. Anti-cytochrome c antibody stainings (BD Biosciences at 1/400 dilution) in larvae expressing Act5C-Gal4 UAS-mitoGFP, 4 days (delg613/+) or 6 days after egg deposition (delg613/Df(3R)ro80b). For the gut, pictures were taken close to the proventiculus. For salivary glands, pictures were taken close to the distal tip. For tracheae, only the GFP channel is shown, and images was taken from the dorsal trunk close to the posterior spiracles. Bar equals 20 µm. (4.30 MB TIF) [file pone.0006935.s002.tif]
