# Supplementary material for: Nutrition Controls Mitochondrial Biogenesis in the Drosophila Adipose Tissue through Delg and Cyclin D/Cdk4
Source: PLoS One. 2009 Sep 9;4(9):e6935. doi: 10.1371/journal.pone.0006935 (PMC2735006; doi:10.1371/journal.pone.0006935)
Supplement: Table S1 — Microarray data show reduced expression of ∼55% of all genes encoding mitochondrial proteins. Fat bodies from wandering heterozygous control animals (+/Df(3R)ro80b) and delg mutants (delg613/Df(3R)ro80b) were dissected, and mRNA levels of all annotated genes were analyzed using the microarray technique (see Materials and Methods). Mitochondrial proteins were clustered as in (Sardiello et al. 2003). Shown are expression levels in the delg mutant, normalized to the expression in control animals. Shown are log2 values of three biological replicates. N/A: Not detected. Reference Sardiello, M., Licciulli, F., Catalano, D., Attimonelli, M., and Caggese, C. 2003. MitoDrome: a database of Drosophila melanogaster nuclear genes encoding proteins targeted to the mitochondrion. Nucleic Acids Res 31(1): 322–324. (0.34 MB DOC) [file pone.0006935.s003.doc]

Table S1. Microarray data show reduced expression of ~55% of all genes encoding mitochondrial proteins. Fat bodies from wandering heterozygous control animals (+/Df(3R)ro^80b^) and *delg* mutants (*delg^613^*/Df(3R)ro^80b^) were dissected, and mRNA levels of all annotated genes were analyzed using the microarray technique (see Materials and Methods). Mitochondrial proteins were clustered as in (Sardiello et al. 2003). Shown are expression levels in the *delg* mutant, normalized to the expression in control animals. Shown are log2 values of three biological replicates. N/A: Not detected.

| **Category/protein name** | ***Drosophila*** | **Mutant/control** | **P Value** |
| --- | --- | --- | --- |
|  |  | **log2 Ratio** |  |
| **Oxidative Phosphorylation** |  |  |  |
| **Complex I: NADH ubiquinone oxidoreductase** |  |  |  |
| 13 KDA-A SUBUNIT | CG8680 | -0.396 | 0.2459 |
| 13 KDA-B SUBUNIT | CG6463 | #N/A | #N/A |
| 15 KDA SUBUNIT | CG11455 | -0.4815 | 0.2521 |
| 18 KDA SUBUNIT | CG12203 | -0.8432 | 0.06985 |
| 19 KDA SUBUNIT | CG3683 | -0.6619 | 0.2307 |
| 20 KDA SUBUNIT | CG9172 | -0.7557 | 0.1645 |
| 20 KDA SUBUNIT | CG2014 | -0.542 | 0.0377 |
| 23 KDA SUBUNIT | ND23 | -0.7564 | 0.128 |
| 24 KDA SUBUNIT | CG5703 | -1.056 | 0.08676 |
| 24 KDA SUBUNIT | CG6485 | #N/A | #N/A |
| 30 KDA SUBUNIT | CG12079 | -0.7189 | 0.167 |
| 39 KDA SUBUNIT | CG6020 | -0.7529 | 0.09105 |
| 42 KDA SUBUNIT | ND42 | -0.8952 | 0.1209 |
| 49 KDA SUBUNIT | CG1970 | -1.097 | 0.04744 |
| 49 KDA SUBUNIT | CG11913 | -0.7146 | 0.3157 |
| 51 KDA SUBUNIT | CG9140 | -0.7098 | 0.1433 |
| 51 KDA SUBUNIT | CG8102 | #N/A | #N/A |
| 75 KDA SUBUNIT | ND75 | -1.147 | 0.05449 |
| ASHI SUBUNIT | CG3192 | -0.1674 | 0.0557 |
| B12 SUBUNIT | CG10320 | #N/A | #N/A |
| B14 SUBUNIT | CG7712 | -0.5509 | 0.2074 |
| B15 SUBUNIT | CG12859 | -0.4983 | 0.221 |
| B17 SUBUNIT | CG13240 | #N/A | #N/A |
| B18 SUBUNIT | CG5548 | -0.495 | 0.2681 |
| B22 SUBUNIT | CG9306 | -0.7567 | 0.1884 |
| B8 SUBUNIT | CG15434 | #N/A | #N/A |
| MLRQ SUBUNIT | CG32230 | -0.5546 | 0.1462 |
| MNLL SUBUNIT | CG18624 | -0.6248 | 0.2441 |
| PDSW SUBUNIT | Pdsw | -0.5835 | 0.3607 |
| SGDH SUBUNIT | l(3)neo18 | -1.114 | 0.074 |
| B14.5A SUBUNIT | CG3621 | -0.4911 | 0.3366 |
| B14.5B SUBUNIT | CG12400 | -0.2631 | 0.5776 |
| B16.6 SUBUNIT | CG3446 | -0.4875 | 0.2457 |
| B17.2 SUBUNIT | CG3214 | -0.761 | 0.2001 |
| ACYL CARRIER PROTEIN | mtacp1 | -0.971 | 0.1577 |
|  |  |  |  |
| **Complex II: Succinate dehydrogenase** |  |  |  |
| FLAVOPROTEIN SUBUNIT | Scs-fp | -1.816 | 0.003488 |
| IRON-SULFUR PROTEIN | SdhB | -0.6526 | 0.2654 |
| CYTOCHROME B560 SUBUNIT | CG6666 | -0.6433 | 0.2212 |
| CYTOCHROME B SMALL SUBUNIT | CG10219 | -0.7886 | 0.1198 |
|  |  |  |  |
| **Complex III: Ubiquinol-cytochrome C oxidoreductase** |  |  |  |
| CYTOCHROME C1, HEME PROTEIN | CG4769 | -0.8994 | 0.123 |
| CYTOCHROME C1, HEME PROTEIN | CG14508 | -1.238 | 0.07241 |
| 11 KDA PROTEIN | Ucrh | #N/A | #N/A |
| 14 KDA PROTEIN | CG3560 | -0.502 | 0.3156 |
| 14 KDA PROTEIN | CG17856 | #N/A | #N/A |
| 6.4 KDA PROTEIN | CG14482 | -0.8167 | 0.2051 |
| 7.2 KDA PROTEIN | ox | -0.7857 | 0.1602 |
| CORE PROTEIN I | CG3731 | -0.9241 | 0.09397 |
| CORE PROTEIN 2 | CG4169 | -1.249 | 0.04613 |
| UBIQUINONE-BINDING PROTEIN QP-C | CG7580 | -0.4777 | 0.1292 |
| IRON-SULFUR SUBUNIT | RFeSP | -1.249 | 0.04231 |
|  |  |  |  |
| **Complex IV: Cytochrome c oxidase** |  |  |  |
| SUBUNIT IV | CG10396 | -0.3509 | 0.6123 |
| SUBUNIT IV | CG10664 | -0.5825 | 0.2506 |
| POLYPEPTIDE VA | CoVa | -1.028 | 0.09908 |
| POLYPEPTIDE VB | CG11015 | -0.8718 | 0.1737 |
| POLYPEPTIDE VB | CG11043 | -0.468 | 0.4649 |
| POLYPEPTIDE VIA | CG17280 | -0.4099 | 0.3331 |
| POLYPEPTIDE VIB | CG18809 | 0.1916 | 0.2611 |
| POLYPEPTIDE VIC | cype | -1.021 | 0.04042 |
| POLYPEPTIDE VIIA-HEART | CG9603 | -0.643 | 0.1543 |
| POLYPEPTIDE VIIA-HEART | CG18193 | #N/A | #N/A |
| POLYPEPTIDE VIIC | CG2249 | -0.7404 | 0.2211 |
|  |  |  |  |
| **Complex V: F0/F1 ATP synthase** |  |  |  |
| ALPHA CHAIN | blw | -1.229 | 0.009389 |
| B CHAIN | ATPsyn-b | -0.9136 | 0.05936 |
| BETA CHAIN | ATPsyn-beta | -1.127 | 0.01272 |
| BETA CHAIN | CG5389 | -1.543 | 0.02287 |
| COUPLING FACTOR 6 | ATPsyn-Cf6 | #N/A | #N/A |
| D CHAIN | ATPsyn-d | #N/A | #N/A |
| DELTA CHAIN | CG2968 | #N/A | #N/A |
| E CHAIN | CG3321 | -0.6795 | 0.1662 |
| EPSILON CHAIN | sun | #N/A | #N/A |
| EPSILON CHAIN | CG12810 | #N/A | #N/A |
| F CHAIN | CG4692 | -0.9272 | 0.1323 |
| G CHAIN | l(2)06225 | -0.5863 | 0.2808 |
| GAMMA CHAIN | ATPsyn-gamma | -0.9118 | 0.0727 |
| LIPID-BINDING PROTEIN P1,P2,P3 | CG1746 | -0.8691 | 0.07243 |
| OLIGOMYCIN SENSITIVITY CONFERRAL PROTEIN | Oscp | -1.057 | 0.03698 |
|  |  |  |  |
| **Others** |  |  |  |
| ATP SYNTHASE COUPLING FACTOR B | CG10731 | #N/A | #N/A |
| COMPLEX I INTERMEDIATE-ASSOCIATED PROTEIN 30 | CG7598 | -0.2817 | 0.03452 |
| CYTOCHROME C | Cyt-c-p | -1.126 | 0.32 |
| CYTOCHROME C | Cyt-c-d | -0.8379 | 0.2723 |
| CYTOCHROME C OXIDASE ASSEMBLY PROTEIN COX11 | CG6922 | #N/A | #N/A |
| CYTOCHROME C OXIDASE COPPER CHAPERONE | CG9065 | 0.1612 | 0.7498 |
| CYTOCHROME OXIDASE BIOGENESIS PROTEIN OXA1 | CG6404 | -1.23 | 0.06769 |
| ELECTRON TRANSFER FLAVOPROTEIN ALPHA-SUBUNIT | wal | -0.3473 | 0.231 |
| ELECTRON TRANSFER FLAVOPROTEIN BETA-SUBUNIT | CG7834 | -0.2171 | 0.48 |
| ELECTRON TRANSFER FLAVOPROTEIN BETA-SUBUNIT | CG12140 | -1.155 | 0.000544 |
| PROTOHEME IX FARNESYLTRANSFERASE | CG5037 | -0.9729 | 0.1654 |
| SCO1, SCO2 PROTEIN HOMOLOG | CG8885 | -0.5594 | 0.1872 |
| SURFEIT LOCUS PROTEIN 1 | Surf1 | -0.4387 | 0.1003 |
|  |  |  |  |
| **Carbohydrate metabolism** |  |  |  |
|  |  |  |  |
| **Tricarboxylic-acid pathway** |  |  |  |
| ACONITATE HYDRATASE | Acon | -0.5247 | 0.2508 |
| ACONITATE HYDRATASE | CG4706 | #N/A | #N/A |
| CITRATE SYNTHASE | CG3861 | #N/A | #N/A |
| CITRATE SYNTHASE | CG14740 | -0.2675 | 0.7174 |
| DIHYDROLIPOAMIDE DEHYDROGENASE | CG7430 | -1.227 | 0.04345 |
| DIHYDROLIPOAMIDE SUCCINYLTRANSFERASE COMPONENT OF 2-OXOGLUTARATE DEHYDROGENASE COMPLEX | CG5214 | -1.085 | 0.04345 |
| FUMARATE HYDRATASE | CG4094 | #N/A | #N/A |
| FUMARATE HYDRATASE | CG4095 | -0.2974 | 0.2956 |
| FUMARATE HYDRATASE | CG31874 | #N/A | #N/A |
| ISOCITRATE DEHYDROGENASE [NAD] SUBUNIT ALPHA | CG12233 | #N/A | #N/A |
| ISOCITRATE DEHYDROGENASE [NAD] SUBUNIT BETA | CG6439 | -0.9005 | 0.01779 |
| ISOCITRATE DEHYDROGENASE [NAD] SUBUNIT GAMMA | CG5028 | -1103 | 0.01309 |
| ISOCITRATE DEHYDROGENASE [NADP] | CG7176 | #N/A | #N/A |
| MALATE DEHYDROGENASE | CG7998 | -0.662 | 0.001544 |
| MALATE DEHYDROGENASE | CG10749 | #N/A | #N/A |
| MALATE DEHYDROGENASE | CG10748 | -1.051 | 0.0837 |
| 2-OXOGLUTARATE DEHYDROGENASE E1 COMPONENT | CG11661 | #N/A | #N/A |
| 2-OXOGLUTARATE DEHYDROGENASE E1 COMPONENT | CG7934 | #N/A | #N/A |
| SUCCINATE DEHYDROGENASE [UBIQUINONE] CYTOCHROME B SMALL SUBUNIT | CG10219 | -0.7886 | 0.1198 |
| SUCCINATE DEHYDROGENASE [UBIQUINONE] FLAVOPROTEIN SUBUNIT | Scs-fp | -1.816 | 0.003488 |
| SUCCINATE DEHYDROGENASE [UBIQUINONE] IRON-SULFUR PROTEIN | SdhB | -0.6526 | 0.2654 |
| SUCCINATE DEHYDROGENASE CYTOCHROME B560 SUBUNIT | CG6666 | -0.6433 | 0.2212 |
| SUCCINYL-COA LIGASE [GDP-FORMING] ALPHA-CHAIN | Scsalpha | 0.2876 | 0.0408 |
| SUCCINYL-COA LIGASE [GDP-FORMING] ALPHA-CHAIN | CG6255 | -0.732 | 0.1449 |
| SUCCINYL-COA LIGASE [GDP-FORMING] BETA-CHAIN | Sucb | -0.9616 | 0.0005897 |
| SUCCINYL-COA LIGASE [ADP-FORMING] BETA-CHAIN | CG11963 | -0.2148 | 0.08904 |
|  |  |  |  |
| **Pyruvate metabolism** |  |  |  |
| ACETYL-COA ACETYLTRANSFERASE | CG10932 | -1.382 | 0.05311 |
| ALDEHYDE DEHYDROGENASE | CG3752 | #N/A | #N/A |
| ALDEHYDE DEHYDROGENASE | CG6309 | #N/A | #N/A |
| DIHYDROLIPOAMIDE ACETYLTRANSFERASE COMPONENT OF PYRUVATE DEHYDROGENASE COMPLEX | CG5261 | -1.466 | 0.06993 |
| DIHYDROLIPOAMIDE DEHYDROGENASE | CG7430 | -1.227 | 0.04345 |
| NADP-DEPENDENT MALIC ENZYME | Mdh | -1.731 | 0.007669 |
| PHOSPHOENOLPYRUVATE CARBOXYKINASE [GTP] | pepck | 0.6376 | 0.4317 |
| PHOSPHOENOLPYRUVATE CARBOXYKINASE [GTP] | CG10924 | 1.368 | 0.1291 |
| PYRUVATE CARBOXYLASE | CG1516 | -0.933 | 0.001057 |
| PYRUVATE DEHYDROGENASE E1 COMPONENT ALPHA SUBUNIT | CG7010 | #N/A | #N/A |
| PYRUVATE DEHYDROGENASE E1 COMPONENT BETA SUBUNIT | CG11876 | -1.802 | 0.01451 |
| PYRUVATE DEHYDROGENASE [LIPOAMIDE]] KINASE ISOZYME 1, 2, 3, 4 | Pdk | -0.3787 | 0.4202 |
| PYRUVATE DEHYDROGENASE [LIPOAMIDE]]-PHOSPHATASE 1, 2 | CG12151 | #N/A | #N/A |
|  |  |  |  |
|  |  |  |  |
| **Others** |  |  |  |
| BIFUNCTIONAL METHYLENETETRAHYDROFOLATE DEHYDROGENASE/CYCLOHYDROLASE | Nmdmc | 2.243 | 0.000292 |
|  |  |  |  |
|  |  |  |  |
| **Amino acid metabolism** |  |  |  |
|  |  |  |  |
| 2-AMINO-3-KETOBUTYRATE COENZYME A LIGASE | CG10361 | #N/A | #N/A |
| 4-AMINOBUTYRATE AMINOTRANSFERASE | CG7433 | #N/A | #N/A |
| 5-AMINOLEVULINIC ACID SYNTHASE | Alas | -0.8496 | 0.113 |
| ACETYL-COA ACETYLTRANSFERASE | CG10932 | -1.382 | 0.05311 |
| 3-KETOACYL-COA THIOLASE | yip2 | 0.4095 | 0.4807 |
| ALDEHYDE DEHYDROGENASE | CG3752 | #N/A | #N/A |
| ALDEHYDE DEHYDROGENASE | CG6309 | #N/A | #N/A |
| AMINOMETHYLTRANSFERASE | CG6415 | -0.4085 | 0.04313 |
| ARGINASE II | arg | -0.927 | 0.01972 |
| ASPARTATE AMINOTRANSFERASE | CG4233 | #N/A | #N/A |
| ASPARTATE AMINOTRANSFERASE | CG8430 | #N/A | #N/A |
| BRANCHED-CHAIN AMINO ACID AMINOTRANSFERASE | CG1673 | -1.41 | 0.01906 |
| CARNITINE O-ACETYLTRANSFERASE | CG5265 | -0.009213 | 0.9894 |
| DELTA-1-PYRROLINE-5-CARBOXYLATE DEHYDROGENASE | CG7145 | -1.072 | 0.05826 |
| DELTA-1-PYRROLINE-5-CARBOXYLATE DEHYDROGENASE | CG6661 | -0.3057 | 0.6511 |
| DIHYDROLIPOAMIDE DEHYDROGENASE | CG7430 | -1.227 | 0.04345 |
| DIHYDROLIPOAMIDE SUCCINYLTRANSFERASE COMPONENT OF 2-OXOGLUTARATE DEHYDROGENASE COMPLEX | CG5214 | -1.085 | 0.04345 |
| ENOYL-COA HYDRATASE | CG6543 | -1409 | 0.02375 |
| GLUTAMATE DEHYDROGENASE | Gdh | -1.33 | 0.0008998 |
| GLUTAMATE DEHYDROGENASE | CG4434 | -0.4843 | 0.5345 |
| GLUTAMINASE, ISOFORMS | nemy | -0.6366 | 0.1185 |
| GLUTARYL-COA DEHYDROGENASE | CG9547 | -0.1829 | 0.3312 |
| GLUTATHIONE S-TRANSFERASE | Mgstl | -1.832 | 0.002797 |
| GLUTATHIONE S-TRANSFERASE | CG12628 | #N/A | #N/A |
| GLYCINE CLEAVAGE SYSTEM H PROTEIN | ppl | 0.2891 | 0.009316 |
| GLYCINE DEHYDROGENASE [DECARBOXYLATING] | CG3999 | 0.4359 | 0.07381 |
| 3-HYDROXYISOBUTYRATE DEHYDROGENASE | CG15093 | -3.25 | 0.01834 |
| HYDROXYMETHYLGLUTARYL-COA LYASE | CG10399 | -1.487 | 0.02191 |
| ISOCITRATE DEHYDROGENASE [NADP] | CG7176 | #N/A | #N/A |
| ISOVALERYL-COA DEHYDROGENASE | CG6638 | -0.5753 | 0.3243 |
| LIPOAMIDE ACYLTRANSFERASE COMPONENT OF BRANCHED-CHAIN ALPHA-KETO ACID DEHYDROGENASE COMPLEX | CG5599 | -2.155 | 0.09607 |
| METHYLCROTONYL-COA CARBOXYLASE ALPHA CHAIN | CG2118 | -0.7907 | 0.2147 |
| METHYLCROTONYL-COA CARBOXYLASE ALPHA CHAIN | CG3267 | -1.311 | 0.1344 |
| METHYLMALONATE-SEMIALDEHYDE DEHYDROGENASE [ACYLATING] | CG17896 | -1.133 | 0.02603 |
| ORNITHINE AMINOTRANSFERASE | CG8782 | #N/A | #N/A |
| 2-OXOGLUTARATE DEHYDROGENASE E1 COMPONENT | CG11661 | #N/A | #N/A |
| 2-OXOGLUTARATE DEHYDROGENASE E1 COMPONENT | CG7934 | #N/A | #N/A |
| 2-OXOISOVALERATE DEHYDROGENASE ALPHA SUBUNIT | CG8199 | -1.676 | 0.1011 |
| 2-OXOISOVALERATE DEHYDROGENASE BETA SUBUNIT | CG17691 | #N/A | #N/A |
| PHOSPHOLIPID HYDROPEROXIDE GLUTATHIONE PEROXIDASE | CG12013 | #N/A | #N/A |
| PROLINE OXIDASE | slgA | -0.4361 | 0.1158 |
| SERINE HYDROXYMETHYLTRANSFERASE | CG3011 | 1.271 | 0.001079 |
| SUCCINATE SEMIALDEHYDE DEHYDROGENASE | CG4685 | -0.3416 | 0.1412 |
| TRIFUNCTIONAL ENZYME ALPHA SUBUNIT | CG4389 | -0.3808 | 0.1103 |
| TRIFUNCTONAL ENZYME BETA SUBUNIT | thiolase | -0.7046 | 0.05069 |
|  |  |  |  |
|  |  |  |  |
| **Metabolism of complex lipids** |  |  |  |
| **Glycerolipid metabolism** |  |  |  |
|  |  |  |  |
| GLYCEROL-3-PHOSPHATE ACYLTRANSFERASE | CG5508 | -0.07336 | 0.8446 |
| GLYCEROL-3-PHOSPHATE DEHYDROGENASE | CG8256 | #N/A | #N/A |
| GLYCEROL-3-PHOSPHATE DEHYDROGENASE | CG2137 | #N/A | #N/A |
|  |  |  |  |
|  |  |  |  |
| **Fatty-acid biosynthesis** |  |  |  |
| ACETYL-COA ACETYLTRANSFERASE | CG10932 | -1.382 | 0.05311 |
| 3-KETOACYL-COA THIOLASE | yip2 | 0.4095 | 0.4807 |
| ENOYL-COA HYDRATASE | CG6543 | -1.409 | 0.02375 |
| TRIFUNCTIONAL ENZYME ALPHA SUBUNIT | CG4389 | -0.3808 | 0.1103 |
| TRIFUNCTONAL ENZYME BETA SUBUNIT | thiolase | -0.7046 | 0.05069 |
|  |  |  |  |
| **Fatty acid metabolism** |  |  |  |
| ACYL-COA DEHYDROGENASE, MEDIUM-CHAIN SPECIFIC | CG12262 | -0.1846 | 0.7489 |
| ACYL-COA DEHYDROGENASE, SHORT/BRANCHED CHAIN SPECIFIC | CG3902 | -2.465 | 0.002935 |
| ACYL-COA DEHYDROGENASE, SHORT-CHAIN SPECIFIC | Arc42 | -0.3312 | 0.03907 |
| ACYL-COA DEHYDROGENASE, SHORT-CHAIN SPECIFIC | CG4860 | 0.09505 | 0.7619 |
| ACYL-COA DEHYDROGENASE, VERY-LONG-CHAIN SPECIFIC | CG7461 | -0.9275 | 0.0009567 |
| ALDEHYDE DEHYDROGENASE | CG3752 | #N/A | #N/A |
| ALDEHYDE DEHYDROGENASE | CG6309 | #N/A | #N/A |
| ALPHA-METHYLACYL-COA RACEMASE | CG9319 | -0.05688 | 0.765 |
| CARNITINE O-PALMITOYLTRANSFERASE I | CPTI | -0.1089 | 0.7989 |
| CARNITINE O-PALMITOYLTRANSFERASE II | CG2107 | -1.008 | 0.007707 |
| DELTA3,5-DELTA2,4-DIENOYL-COA ISOMERASE | CG9577 | 0.3199 | 0.49 |
| GLUTARYL-COA DEHYDROGENASE | CG9547 | -0.1829 | 0.3312 |
| 3,2-TRANS-ENOYL-COA ISOMERASE | CG4598 | -0.5288 | 0.01024 |
|  | CG4594 | -0.4567 | 0.1352 |
|  |  |  |  |
| **Others** |  |  |  |
| ADRENODOXIN | CG1319 | -0.4866 | 0.2691 |
| SUCCINYL-COA:3-KETOACID-COENZYME A TRANSFERASE | CG1140 | -2.14 | 0.02452 |
|  |  |  |  |
|  |  |  |  |
| **Nucleotide metabolism** |  |  |  |
|  |  |  |  |
| ADENYLATE KINASE ISOENZYME 2 (4) | Adk2 | -1074 | 0.0695 |
| GTP:AMP PHOSPHOTRANSFERASE | Adk3 | -0.9146 | 0.009351 |
| DIHYDROOROTATE DEHYDROGENASE | Dhod | -0.2343 | 0.2378 |
| THYMIDINE KINASE 2 | dnk | -0.3524 | 0.5754 |
|  |  |  |  |
| **Sulfur metabolism** |  |  |  |
|  |  |  |  |
| CYSTEINE DESULFURASE | CG12264 | 0.7076 | 0.01839 |
| SULFITE OXIDASE | CG7280 | -0.574 | 0.07943 |
|  |  |  |  |
|  |  |  |  |
| **Metabolism of Cofactors and Vitamins** |  |  |  |
|  |  |  |  |
| COPROPORPHYRINOGEN III OXIDASE | Coprox | -0.5077 | 0.01181 |
| CYTOCHROME C-TYPE HEME LYASE | CG6022 | #N/A | #N/A |
| DELTA 1-PYRROLINE-5-CARBOXYLATE SYNTHETASE | CG7470 | -0.228 | 0.2765 |
| FERROCHELATASE | ferrochelatase | 0.1054 | 0.5482 |
| FOLYLPOLYGLUTAMATE SYNTHASE | folc | #N/A | #N/A |
| NADPH:ADRENODOXIN OXIDOREDUCTASE | dare | -0.6352 | 0.07775 |
| PROTOPORPHYRINOGEN OXIDASE | CG5796 | #N/A | #N/A |
|  |  |  |  |
| **DNA and RNA** |  |  |  |
|  |  |  |  |
| ATP-BINDING CASSETTE SUB-FAMILY E MEMBER 1 | CG5651 | 0.3618 | 0.05848 |
| DNA POLYMERASE GAMMA, SUBUNIT 1 | tam | #N/A | #N/A |
| DNA POLYMERASE GAMMA SUBUNIT 2 | CG8969 | #N/A | #N/A |
| DNA-DIRECTED RNA POLYMERASE | CG4644 | #N/A | #N/A |
| ENDONUCLEASE G | CG8862 | -0.2413 | 0.6199 |
| OLIGORIBONUCLEASE | CG10214 | 0.09854 | 0.8419 |
| SINGLE-STRANDED DNA-BINDING PROTEIN | mtSSB | 0.1612 | 0.7555 |
| TRANSCRIPTION FACTOR 1 | TFAM | 0.1236 | 0.5492 |
|  |  |  |  |
| **Protein synthesis** |  |  |  |
|  |  |  |  |
| **Ribosomal proteins** |  |  |  |
| 28S RIBOSOMAL PROTEIN S6 | mRpS6 | #N/A | #N/A |
| 28S RIBOSOMAL PROTEIN S12 | tko | -0.403 | 0.5336 |
| 28S RIBOSOMAL PROTEIN S15 | bonsai | -0.6215 | 0.3244 |
| 28S RIBOSOMAL PROTEIN S16 | mRpS16 | #N/A | #N/A |
| 28S RIBOSOMAL PROTEIN S17 | mRpS17 | -0.05698 | 0.8577 |
| 28S RIBOSOMAL PROTEIN S21 | mRpS21 | -0.232 | 0.4362 |
| 28S RIBOSOMAL PROTEIN S22 | mRpS22 | -0.5789 | 0.2026 |
| 28S RIBOSOMAL PROTEIN S25 | mRpS25 | -0.4499 | 0.4929 |
| 28S RIBOSOMAL PROTEIN S29 | mRpS29 | -0.733 | 0.1192 |
| 60S RIBOSOMAL PROTEIN L3 | mRpL3 | -0.6491 | 0.2356 |
| 60S RIBOSOMAL PROTEIN L7/L12 | mRpL7-L12 | #N/A | #N/A |
|  |  |  |  |
| **Others** |  |  |  |
| ELONGATION FACTOR TS, MITOCHONDRIAL | CG6412 | -0.5509 | 0.1366 |
| ELONGATION FACTOR TU, MITOCHONDRIAL | EfTuM | -0.401 | 0.5277 |
| MITOCHONDRIAL PEPTIDE CHAIN RELEASE FACTOR 1 | CG5705 | -0.6809 | 0.06397 |
| PROBABLE GLUTAMYL-TRNA(GLN) AMIDOTRANSFERASE SUBUNIT B, MITOCHONDRIAL | CG5463 | -0.2724 | 0.3364 |
| PROBABLE LEUCYL-TRNA SYNTHETASE, MITOCHONDRIAL | CG7479 | #N/A | #N/A |
| TRANSLATION INITIATION FACTOR IF-2, MITOCHONDRIAL | CG12413 | -0.4218 | 0.2549 |
|  |  |  |  |
| **Protein destination** |  |  |  |
|  |  |  |  |
| **Protein folding and stabilization** |  |  |  |
| GRPE PROTEIN HOMOLOG 2, MITOCHONDRIAL | Roe1 | -0.609 | 0.2657 |
| 10 KDA HEAT SHOCK PROTEIN, MITOCHONDRIAL | CG11267 | 0.1119 | 0.8877 |
| 10 KDA HEAT SHOCK PROTEIN, MITOCHONDRIAL | CG9920 | -0.4378 | 0.5526 |
| 60 KDA HEAT SHOCK PROTEIN, MITOCHONDRIAL | Hsp60 | -0.8155 | 0.2599 |
| 60 KDA HEAT SHOCK PROTEIN, MITOCHONDRIAL | CG7235 | #N/A | #N/A |
| 60 KDA HEAT SHOCK PROTEIN, MITOCHONDRIAL | Hsp60B | 0.1648 | 0.803 |
| 60 KDA HEAT SHOCK PROTEIN, MITOCHONDRIAL | CG16954 | #N/A | #N/A |
| MITOCHONDRIAL STRESS-70 PROTEIN | Hsc70-5 | -0.6826 | 0.1484 |
| MITOCHONDRIAL STRESS-70 PROTEIN | Hsc70-3 | -0.1761 | 0.1888 |
| MITOCHONDRIAL STRESS-70 PROTEIN | Hsc70-4 | 0.3828 | 0.03648 |
| MITOCHONDRIAL STRESS-70 PROTEIN | Hsp68 | -1041 | 0.04477 |
| MITOCHONDRIAL STRESS-70 PROTEIN | Hsc70-1 | #N/A | #N/A |
| MITOCHONDRIAL STRESS-70 PROTEIN | Hsc70-2 | -0.5638 | 0.498 |
|  |  |  |  |
|  |  |  |  |
| **Protein targeting, sorting and translocation** |  |  |  |
| IMPORT INNER MEMBRANE TRANSLOCASE SUBUNIT TIM10 | Tim10 | -0.6052 | 0.1925 |
| IMPORT INNER MEMBRANE TRANSLOCASE SUBUNIT TIM10 | Tim13 | #N/A | #N/A |
| IMPORT INNER MEMBRANE TRANSLOCASE SUBUNIT TIM17 A | Tim17a1 | #N/A | #N/A |
| IMPORT INNER MEMBRANE TRANSLOCASE SUBUNIT TIM17 A | Tim17a2 | #N/A | #N/A |
| IMPORT INNER MEMBRANE TRANSLOCASE SUBUNIT TIM17 B | Tim17b1 | -0.915 | 0.1895 |
| IMPORT INNER MEMBRANE TRANSLOCASE SUBUNIT TIM17 B | Tim17b2 | -0.3585 | 0.64 |
| IMPORT INNER MEMBRANE TRANSLOCASE SUBUNIT TIM17 B | CG1724 | #N/A | #N/A |
| IMPORT INNER MEMBRANE TRANSLOCASE SUBUNIT TIM22 | CG31229 | -0.4247 | 0.3206 |
| IMPORT INNER MEMBRANE TRANSLOCASE SUBUNIT TIM23 | Tim23 | #N/A | #N/A |
| IMPORT INNER MEMBRANE TRANSLOCASE SUBUNIT TIM8 A | Tim8 | 0.2068 | 0.6615 |
| IMPORT INNER MEMBRANE TRANSLOCASE SUBUNIT TIM9 A | Tim9a | -0.1463 | 0.5236 |
| IMPORT INNER MEMBRANE TRANSLOCASE SUBUNIT TIM9 B | Tim9b | #N/A | #N/A |
| IMPORT INNER MEMBRANE TRANSLOCASE SUBUNIT TIM44 | CG11779 | -0.7659 | 0.1618 |
| METAXIN 1 | CG9393 | 1.58 | 0.0006938 |
| METAXIN 2 | CG8004 | 0.03546 | 0.8628 |
| METAXIN 2 | CG5662 | #N/A | #N/A |
| MITOCHONDRIAL IMPORT RECEPTOR SUBUNIT TOM7 | CG8226 | #N/A | #N/A |
| IMPORT RECEPTOR SUBUNIT TOM20 | CG7654 | #N/A | #N/A |
| IMPORT RECEPTOR SUBUNIT TOM20 | CG14690 | #N/A | #N/A |
| MITOCHONDRIAL IMPORT RECEPTOR SUBUNIT TOM40 | Tom40 | -0.5994 | 0.4031 |
| MITOCHONDRIAL IMPORT RECEPTOR SUBUNIT TOM40 | CG8330 | #N/A | #N/A |
| MITOCHONDRIAL PRECURSOR PROTEINS IMPORT RECEPTOR | CG6756 | #N/A | #N/A |
|  |  |  |  |
| **Proteolysis** |  |  |  |
| AFG3-LIKE PROTEIN 2 | CG6512 | 0.348 | 0.1676 |
| INTERMEDIATE PEPTIDASE, MITOCHONDRIAL | CG7791 | -0.4867 | 0.1496 |
| LON PROTEASE HOMOLOG, MITOCHONDRIAL | CG8798 | -0.1648 | 0.6546 |
| MITOCHONDRIAL PROCESSING PEPTIDASE ALPHA SUBUNIT | CG8728 | -0.4287 | 0.2391 |
| MITOCHONDRIAL PROCESSING PEPTIDASE BETA SUBUNIT | CG3731 | -0.9241 | 0.09397 |
| PARAPLEGIN | CG2658 | -0.1554 | 0.6124 |
| ATP-DEPENDENT CLP PROTEASE ATP-BINDING SUBUNIT CLPX, MITOCHONDRIAL | CG4538 | 0.8964 | 0.00109 |
| PUTATIVE ATP-DEPENDENT CLP PROTEASE PROTEOLYTIC SUBUNIT, MITOCHONDRIAL | CG5045 | -0.06721 | 0.4153 |
|  |  |  |  |
| **Transport facilitation** |  |  |  |
|  |  |  |  |
| ADP, ATP CARRIER PROTEIN | sesB | -1.814 | 0.01599 |
| ADP, ATP CARRIER PROTEIN | Ant2 | -1.234 | 0.01651 |
| ATP-BINDING CASSETTE, SUB-FAMILY B, MEMBER 10 | CG3156 | -1.099 | 0.05549 |
| ATP-BINDING CASSETTE, SUB-FAMILY B, MEMBER 6 | CG4225 | -0.9841 | 0.03542 |
| ATP-BINDING CASSETTE, SUB-FAMILY B, MEMBER 7 | CG7955 | -0.8648 | 0.03067 |
| ATP-BINDING CASSETTE, SUB-FAMILY B, MEMBER 8 | CG1824 | -0.516 | 0.2268 |
| CALCIUM-BINDING CARRIER PROTEIN ARALAR1-2 | Aralar1 | 0.5779 | 0.312 |
| CARNITINE/ACYLCARNITINE CARRIER PROTEIN | colt | -0.7106 | 0.05154 |
| CARNITINE/ACYLCARNITINE CARRIER PROTEIN | CG3476 | -0.3467 | 0.3661 |
| FRATAXIN | fh | -0.1825 | 0.4495 |
| FOLATE TRANSPORTER/CARRIER | CG8026 | -0.8916 | 0.006783 |
| ORNITHINE TRANSPORTER 1 | CG1628 | -0.7273 | 0.251 |
| NONSPECIFIC LIPID-TRANSFER PROTEIN | Scpx | -0.7411 | 0.1504 |
| 2-OXOGLUTARATE/MALATE CARRIER PROTEIN | CG1907 | -0.6005 | 0.0141 |
| 2-OXOGLUTARATE/MALATE CARRIER PROTEIN | CG18418 | -0.00297 | 0.9961 |
| 2-OXOGLUTARATE/MALATE CARRIER PROTEIN | CG7514 | #N/A | #N/A |
| PHOSPHATE CARRIER PROTEIN | CG9090 | -0.5926 | 0.1292 |
| PHOSPHATE CARRIER PROTEIN | Mpcp | -0.9411 | 0.02879 |
| TRICARBOXYLATE TRANSPORT PROTEIN | CG31305 | #N/A | #N/A |
| VOLTAGE-DEPENDENT ANION-SELECTIVE CHANNEL PROTEINS | porin | -0.3118 | 0.3719 |
| VOLTAGE-DEPENDENT ANION-SELECTIVE CHANNEL PROTEINS | CG17137 | #N/A | #N/A |
|  |  |  |  |
| **Cell rescue, defense and cell death** |  |  |  |
|  |  |  |  |
| APOPTOSIS REGULATOR BCL-2 | debcl | -0.1679 | 0.4539 |
| PROGRAMED CELL DEATH PROTEIN 8 | CG7263 | -0.8448 | 0.1818 |
| SUPEROXIDE DISMUTASE [MN] | Sod2 | -1.145 | 0.03136 |
| THIOREDOXIN | CG8517 | -0.1935 | 0.643 |
| THIOREDOXIN | CG8993 | 0.1097 | 0.6919 |
| THIOREDOXIN | CG3719 | -0.0881 | 0.8072 |
| THIOREDOXIN-DEPENDENT PEROXIDE REDUCTASE | Prx5037 | -1.636 | 0.02061 |
|  |  |  |  |
| **Others** |  |  |  |
|  |  |  |  |
| BRAIN MITOCHONDRIAL CARRIER PROTEIN-1 | Bmcp | #N/A | #N/A |
| COMPLEMENT COMPONENT 1, Q SUBCOMPONENT BINDING PROTEIN | CG6459 | -0.3627 | 0.5992 |
| PERIPHERAL-TYPE BENZODIAZEPINE RECEPTOR | CG2789 | 0.4688 | 0.05999 |
| UNCOUPLING PROTEIN 4 | Ucp4A | -0.8242 | 0.03868 |
| UNCOUPLING PROTEIN 4 | Ucp4B | #N/A | #N/A |
| UNCOUPLING PROTEIN 4 | Ucp4C | #N/A | #N/A |

**Reference**

Sardiello, M., Licciulli, F., Catalano, D., Attimonelli, M., and Caggese, C. 2003. MitoDrome: a database of Drosophila melanogaster nuclear genes encoding proteins targeted to the mitochondrion*. Nucleic Acids R*e**s** 31(1): 322-324.
